# Quality, Spending, Utilization, and Outcomes Among Dual-Eligible Medicare-Medicaid Beneficiaries in Integrated Care Programs: A Systematic Review

**DOI:** 10.1001/jamahealthforum.2024.2187

**Published:** 2024-07-19

**Authors:** Eric T. Roberts, Ciara Duggan, Rebekah Stein, Sriya Jonnadula, Kenton J. Johnston, José F. Figueroa

**Affiliations:** 1 University of Pennsylvania Perelman School of Medicine, Philadelphia; 2 University of Pennsylvania Leonard Davis Institute of Health Economics, Philadelphia; 3 Harvard T.H. Chan School of Public Health, Boston, Massachusetts; 4 University of Pennsylvania Wharton School, Philadelphia; 5 Washington University in St Louis, Missouri; 6 Brigham and Women’s Hospital, Boston, Massachusetts

## Abstract

**Importance:**

Most dual-eligible Medicare-Medicaid beneficiaries are enrolled in bifurcated insurance programs that pay for different components of care. Therefore, policymakers are prioritizing expansion of integrated care plans (ICPs) that manage both Medicare and Medicaid benefits and spending.

**Objective:**

To review evidence of the association between ICPs and health care spending, quality, utilization, and patient outcomes among dual-eligible beneficiaries.

**Evidence Review:**

A search was conducted of PubMed/MEDLINE (January 1, 2010, through November 1, 2023) and Google Scholar (January 1, 2010, through October 1, 2023) and augmented with reports from US federal and state government websites. Three categories of ICPs were evaluated: Programs of All-Inclusive Care for the Elderly (PACE), Medicare-Medicaid Plans (MMPs), and Fully Integrated Dual Eligible Special Needs Plans (FIDE-SNPs) and related models aligning Medicare and Medicaid coverage. The review included studies that evaluated beneficiaries dually eligible for and enrolled in full Medicaid; compared an ICP to a nonintegrated arrangement; and evaluated utilization, spending, care coordination, patient experience, or health for 100 or more beneficiaries.

**Findings:**

In all, 26 ICP evaluations met the inclusion criteria and were included in the analysis: 5 of PACE, 13 of MMPs, and 8 of FIDE-SNPs and other aligned models. Evidence generally showed associated reductions in long-term nursing home stays in PACE (3 of 4 studies) and FIDE-SNPs and related aligned models (3 of 5 studies) but was mixed in evaluations of MMPs. Four of 9 studies of MMPs and 2 of 3 studies of FIDE-SNPs found higher outpatient use, although other studies showed no difference. Evidence on Medicaid spending was limited, whereas 8 of 10 studies of MMPs showed an association between these plans and higher Medicare spending. Evidence was mixed or inconclusive regarding care coordination and hospitalizations, and it was insufficient to evaluate patient satisfaction, health, and outcomes in beneficiary subgroups (eg, those with serious mental illness). Furthermore, studies had limited ability to control for bias from unmeasured differences between enrollees of ICPs compared with nonintegrated models.

**Conclusions and Relevance:**

This systematic review found variability and gaps in evidence regarding ICPs and spending, quality, utilization, and outcomes. Studies found some ICPs were associated with reductions in long-term nursing home admissions, and several identified increases in outpatient care. However, MMPs were primarily associated with higher Medicare spending. Evidence for other outcomes was limited or inconclusive. Research addressing these evidence gaps is needed to guide ongoing efforts to integrate coverage and care for dual-eligible beneficiaries.

## Key Points

**Question:**

To what extent are Medicare and Medicaid integrated care plans (ICPs) associated with differences in quality, spending, utilization, and patient outcomes among dual-eligible beneficiaries?

**Findings:**

This systematic review including 26 ICP evaluations found evidence of associations between ICPs and reductions in long-term nursing home stays in 2 of 3 of categories of ICPs and some evidence of ICP association with greater outpatient care. However, for 1 category of ICPs, studies primarily found higher Medicare spending, and across ICP categories, evidence was limited or inconsistent regarding Medicaid spending, hospital admissions, care coordination, patient satisfaction, and health.

**Meaning:**

These findings suggest that despite evidence of some ICPs being associated with outcomes consistent with policymakers’ objectives (eg, reducing nursing home stays), the literature is limited and inconclusive regarding other outcomes.

## Teaser

This systematic review assesses the evidence of the association between integrated care programs and health care quality, spending, utilization, and patient outcomes among Medicare-Medicaid dual-eligible beneficiaries.

## Introduction

US state and federal policymakers are pursuing reforms to promote higher quality and fiscally sustainable care for the nation’s 12.5 million dual-eligible Medicare and Medicaid beneficiaries. A central focus of these reforms has been the expansion of integrated care programs (ICPs), which coordinate care for dual-eligible beneficiaries and bear risk for both Medicare and Medicaid spending.^1,2,3^

ICPs are intended to address concerns that a lack of coordination between Medicare and Medicaid results in poor quality and inefficient care.^4^ For dual-eligible beneficiaries, Medicare (a federal program) is the primary payer for outpatient, hospital, and postacute care, while Medicaid (administered by states) pays for long-term care, including nursing home care and home- and community-based services (HCBS) and some behavioral health care.^5^ However, 90% of dual-eligible beneficiaries are not enrolled in ICPs, meaning that their Medicare- and Medicaid-covered services and spending are managed by separate entities.^6^ Research has shown that a lack of integration hinders care coordination and produces unintended incentives to shift costs between Medicare and Medicaid.^4,7,8,9^ Additionally, there are concerns that nonintegrated care contributes to poor outcomes for beneficiaries with substantial needs, such as those with serious mental illness or receiving long-term services and supports.^10,11^

Policymakers have highlighted 3 distinct categories of ICPs as templates for integrated program expansion: Programs of All-Inclusive Care for the Elderly (PACE), Medicare-Medicaid Plans (MMPs), and Fully Integrated Dual Eligible Special Needs Plans (FIDE-SNPs).^2^ PACE is a managed care program that serves community-dwelling adults 55 years and older who need nursing home−level care, most of whom are dual-eligible beneficiaries.^12^ This program covers all Medicare and Medicaid services and provides supportive services in adult day health centers. MMPs receive blended capitation payments from Medicare and Medicaid. The plans were tested by 10 states starting in 2013 under the Centers for Medicare & Medicaid (CMS) Financial Alignment Demonstration, and continued to operate in 9 states in 2023 (all MMPs are scheduled to end by 2025).^1,2^ FIDE-SNPs are a subset of Dual-Eligible Special Needs Plans (D-SNPs), which are Medicare Advantage (MA) plans that exclusively serve dual-eligible beneficiaries.^2^ Unlike most D-SNPs, which only manage Medicare spending, FIDE-SNPs also bear risk for Medicaid spending through capitation contracts with Medicaid programs, or in some cases, through an affiliated Medicaid managed care plan.^6^

Proposals to expand ICPs have motivated an interest in understanding whether existing programs provide higher quality and efficient care.^3,13^ While there have been prior efforts to track and compile evidence on ICPs,^14,15,16^ to our knowledge, this literature has not been systematically reviewed. Therefore, we systematically reviewed evidence on the association of PACE, MMPs, and FIDE-SNPs with health care use, quality of care, spending, patient-reported experiences, and health outcomes across categories of ICPs and in subpopulations of dual-eligible beneficiaries. We also evaluated the quality of available evidence, including methods to address selection bias that can arise because enrollment in ICPs is voluntary and there may be unmeasured differences between beneficiaries who enroll in ICPs compared with other plans.^16^

## Methods

We conducted this systematic review using the relevant sections of the Preferred Reporting Items for Systematic Reviews and Meta-analyses (PRISMA) reporting guidelines. We developed prespecified search criteria for PubMed and Google Scholar and augmented search results with evaluations published on government websites. The eMethods in Supplement 1 describes our search strategy and inclusion criteria. This review included studies that compared ICPs to nonintegrated arrangements. We included 3 types of ICPs: PACE, MMPs, and FIDE-SNPs and related models that align coverage across Medicare and Medicaid. Other aligned models included the Massachusetts Senior Care Options (SCO)^17^ and Minnesota Senior Health Options (MSHO)^18^ programs, which were precursors to FIDE-SNPs and are now classified as FIDE-SNPs,^2^ and arrangements in which beneficiaries enrolled in companion Medicare and Medicaid managed care plans operated by the same parent insurers (Tennessee^19^ and Oregon^20^). Evaluations of managed fee-for-service plans in Colorado and Washington under the CMS Financial Alignment Demonstration were not included because most proposals to expand ICPs focus on capitated models.^3^

Nonintegrated arrangements were those in which Medicare spending was managed separately from Medicaid and included fee-for-service Medicare, conventional MA plans, and coordination-only D-SNPs.^1^ Coordination-only D-SNPs were rarely assessed as the comparison group, and we did not classify these plans as ICPs because they do not manage Medicaid spending.

We limited this review to studies of dual-eligible beneficiaries with full Medicaid, who are the focus of integration policy because they qualify for all Medicaid-covered services, including long-term care.^2^ We further limited our review to studies with at least 100 participants; that reported quantitative findings for at least 1 quality, spending, utilization, patient experience, or health outcome; and that were published or accepted for publication from January 1, 2010, to October 1, 2023 (Google Scholar), and November 1, 2023 (PubMed and government websites). We incorporated updated findings from 3 federally funded MMP evaluations from December 2023. The Figure shows the PRISMA diagram for study inclusion.

**Figure.**
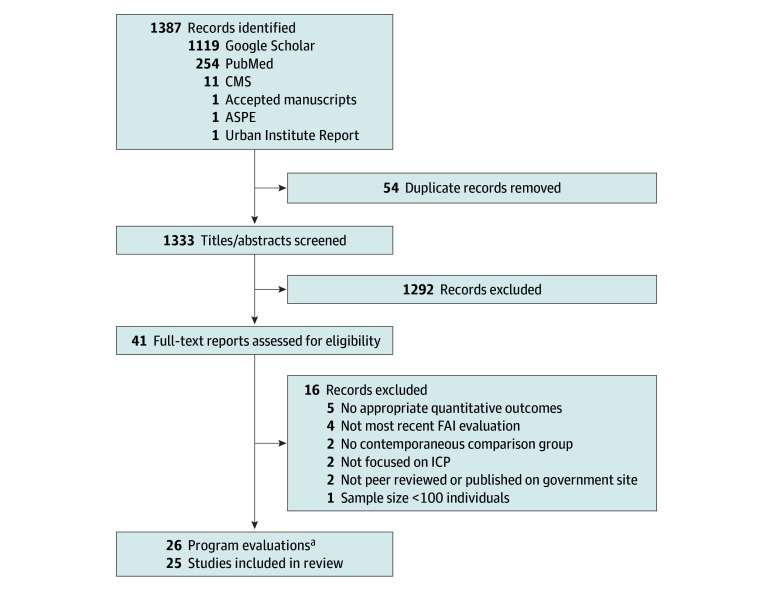
Study Inclusion PRISMA Flow Diagram ^a^In all, 25 unique studies were included in this review: 24 that evaluated assessed a single type of ICP and 1 that assessed both PACE and FIDE-SNPs. For our review, we counted the latter study twice (1 set of findings for each program), producing 26 evaluations: 5 of PACE programs, 13 of MMPs, and 8 of FIDE-SNPs and similar managed care models. For 3 of the MMP evaluations, updated reports were published in December 2023, after our initial screening and data extraction was concluded. We incorporated updated findings from these latest evaluations to reflect the most recent set of reported results. ASPE indicates the US Office of the Assistant Secretary for Planning and Evaluation; CMS, the US Centers for Medicare & Medicaid Services; FAI, Financial Alignment Initiative; FIDE-SNPs, Fully Integrated Dual Eligible Special Needs Plans; ICP, integrated care plan; MMPs, Medicare-Medicaid Plans; and PACE, Programs of All-Inclusive Care for the Elderly.

### Data Abstraction

For each study, 1 author (E.T.R.) abstracted the following information: ICP evaluated; number and age of beneficiaries included scope (single state, several states, or national); research design (observational cross-sectional, observational longitudinal, or quasi-experimental); and findings. This author (E.T.R.) also evaluated the quality of evidence based on how each study addressed selection bias. A second author (C.E.D. or R.S.) independently checked this information (eMethods in Supplement 1 for details).

Based on the literature and conceptual frameworks for integrated coverage, we characterized the expected association between ICPs and spending, quality, utilization, patient experience, and health outcomes.^21,22,23^ These hypothesized associations include, for example, fewer long-term nursing home admissions, increased use of HCBS (ie, community-based long-term services and supports to assist older adults and people with a disability), and better patient-reported experience with health plans. We counted the number of studies whose results were consistent with, contrary to, and mixed or inconclusive regarding these hypothesized findings. Because of differences across ICPs, we reported findings separately for each ICP type and did not conduct a quantitative meta-analysis.

## Results

### Summary of Studies

Twenty-five unique studies were included in this review: 24 that evaluated a single type of ICP and 1 that evaluated both PACE and FIDE-SNPs.^22^ We counted the latter study twice (1 set of findings for each program) for a total of 26 program evaluations: 5 of PACE, 13 of MMPs, and 8 of FIDE-SNPs and other aligned models (Table 1).^12,17,18,19,20,21,22,24,25,26,27,28,29,30,31,32,33,34,35,36,37,38,39,40,41^ Fourteen studies assessed ICPs over periods of less than 5 years (1 of PACE, 7 of MMPs, and 6 of FIDE-SNPs and other aligned models), and 12 assessed study periods of 5 years or longer (4 of PACE, 6 of MMPs, and 2 of FIDE-SNPs and other aligned models). Studies of PACE were limited to beneficiaries 55 years and older and evaluations of Massachusetts’ MMP were limited to those 65 years or younger, reflecting these programs’ eligibility criteria; other studies either included dual-eligible beneficiaries of all ages or only those 65 years and older. Twenty-four studies primarily used administrative data and 2 used survey data.

**Table 1.** Characteristics of Included Studies on Dual-Eligible Medicare-Medicaid Beneficiaries in Integrated Care Programs (ICP)

Source^a^	No. of beneficiaries, state^b^	Primary data source and years	ICP evaluation time, y	Beneficiaries’ age, y	Subgroups evaluated	Design^c^	Quality^d^
**PACE** ^e^							
Chapin et al,^34^ 2013	136 Enrolled, Kansas	Administrative, 2006-2011	5.5	≥65	People eligible for NH-level care^e^; with frailty; with greater cognitive needs; or at end of life	Observational, longitudinal	3
Feng et al,^22^ 2021	25 665 Enrolled, national	Administrative, 2015	1	≥55	People eligible for NH-level care^e^	Observational, cross-sectional	4
Ghosh et al,^12^ 2015	3725 Enrolled, 8 states	Administrative, 2006-2011	5.5	≥66	People eligible for NH-level care^e^	Observational, longitudinal	3
Segelman et al,^36^ 2017	4733 Enrolled, 12 states	Administrative, 2005-2009	3-5	≥55	People eligible for NH-level care^e^	Observational, longitudinal	3
Wieland et al,^35^ 2013	948 Enrolled, South Carolina	Administrative, 1994-2005	11	≥55	People eligible for NH-level care^e^	Observational, longitudinal	3
**MMPs** ^f^							
Caswell et al,^21^ 2023	16 680 eligible, Massachusetts	Administrative, 2016-2018	3	≤64	Not evaluated	Quasi-experimental	3
Chen et al,^39^ 2018	13 370 Enrolled, South Carolina	Administrative, 2011-2016	2	≥65	Not evaluated	Observational, longitudinal	3
Chepaitis et al,^26^ 2021	57 937 Eligible, Virginia	Administrative, 2012-2017	4	≥21^h^	Not evaluated	Quasi-experimental	3
Gattine et al,^28^ 2023	118 443 Eligible, Massachusetts	Administrative, 2011-2019	6	≥21 to ≤64	People receiving LTSS and populations with SPMI	Quasi-experimental	3
Graham et al,^38^ 2018	488 Enrolled, California	Survey, 2016-2017	6-22 mo	≥21	Not evaluated	Observational, cross-sectional	4
Griffin et al,^24^ 2023	141 966 eligible, Ohio	Administrative, 2012-2020	6.5	≥18	People receiving LTSS and populations with SPMI	Quasi-experimental	3
Griffin et al,^33^ 2023	157 348 eligible, Texas	Administrative, 2013-2020	6	≥21	People receiving LTSS and populations with SPMI	Quasi-experimental	3
Holladay et al,^29^ 2022	109 548 eligible	Administrative, 2013-2018	4	≥21	People receiving LTSS and populations with SPMI	Quasi-experimental	3
Holladay et al,^25^ 2022	263 128 eligible	Administrative, 2012-2019	5	≥21	People receiving LTSS and populations with SPMI	Quasi-experimental	3
Howard et al,^27^ 2023	25 410 eligible, South Carolina	Administrative, 2013-2020	6	≥65	People receiving LTSS and populations with SPMI	Quasi-experimental	3
Snow et al,^32^ 2023	22 488 eligible, New York	Administrative, 2014-2020	4.75	≥21	People with intellectual and developmental disabilities	Quasi-experimental	3
Khatutsky et al,^31^ 2023	479 461 eligible, California	Administrative, 2012-2019	6	≥21	Not evaluated	Quasi-experimental	3
Gattine et al,^30^ 2023	37 126 eligible, Rhode Island	Administrative, 2014-2020	4.5	≥21	People receiving LTSS and populations with SPMI	Quasi-experimental	3
**FIDE-SNPs and similar managed care models** ^g^							
Anderson et al,^18^ 2020	99 761 Enrolled, Minnesota	Administrative, 2010-2012	3	≥65	Not evaluated	Observational, cross-sectional	4
Feng et al,^22^ 2021	89 949 Enrolled, national	Administrative, 2015	1	≥21	Not evaluated	Observational, cross-sectional	4
JEN Associates,^17^ 2013	12 064 Enrolled, Massachusetts	Administrative, 2004-2009	6	≥65	Not evaluated	Observational, longitudinal	3
Jung et al,^40^ 2015	1090 Enrolled, Massachusetts	Administrative, 2007-2009	3	≥65	People hospitalized with CHF or COPD	Observational, longitudinal	3
Keohane et al,^19^ 2021	129 731 Eligible, Tennessee	Administrative, 2011-2017	6-7	≥21	People receiving LTSS	Quasi-experimental	3
Kim et al,^20^ 2019	About 17 320 Enrolled	Administrative, 2011-2014	4	≥18	Not evaluated	Observational, longitudinal	3
Meyers et al,^41^ 2023	10 565 Enrolled, national	Survey, 2015-2018	4	≥21	Not evaluated	Observational, cross-sectional	4
Roberts et al,^37^ 2023	7967 Enrolled, Pennsylvania	Administrative, 2015-2020	3	≥21	People eligible for NH-level care	Observational, longitudinal	3

Abbreviations: CHF, congestive heart failure; COPD, chronic obstructive pulmonary disease; FIDE-SNPs, Fully Integrated Dual Eligible Special Needs Plans; LTSS, long-term services and supports; MMPs, Medicare-Medicaid Plans; NH, nursing home; PACE, Programs of All-Inclusive Care for the Elderly; SPMI, serious and persistent mental illness.

^a^ eTable 4 in Supplement 1 provides details on data extracted.

^b^ Either number of people ICP eligible or enrolled, per study design.

^c^ Cross-sectional compared dual-eligible beneficiaries with vs without ICP at a single point in time or pooled over multiple periods; longitudinal examined cohorts over multiple periods or trends in repeated cross-sections; quasi-experimental included difference-in-differences and regression discontinuity designs.

^d^ Rating scale: 1, randomized clinical trial or systematic review with meta-analysis; 2, controlled trial without randomization or prospective comparative cohort trial; 3, case-control or retrospective cohort study; 4, case series with or without intervention or cross-sectional study; 5, opinion of respected authorities or case reports.

^e^ All PACE beneficiaries are eligible for NH-level care.

^f^ Quantitative studies of capitated MMPs tested under the Financial Alignment Demonstration only; excluded managed fee-for-service plans.

^g^ Either fully integrate Medicare and Medicaid spending or have dual-eligible beneficiaries in aligned Medicare and Medicaid managed care plans operated by the same insurer.

### Spending

Eleven studies evaluated Medicare spending (1 of PACE and 10 of MMPs) and 7 studies evaluated Medicaid spending (3 of PACE and 4 of MMPs); neither Medicare nor Medicaid spending was assessed in studies of FIDE-SNPs (Table 2). Eight evaluations found an association of MMPs implementation with increased Medicare spending of $36.98 to $118.05 per person-month during 4.0 to 6.5 years,^24,25,26,27,28,29,30,31^ whereas 2 MMP evaluations and 1 PACE study found no significant difference in Medicare spending.^12,32,33^ Two studies of PACE found lower Medicaid spending,^34,35^ 1 study of PACE and 2 studies of MMPs found an increase in Medicaid spending,^12,28,31^ and 2 studies of MMPs found no difference.^32,33^ Only 1 study of PACE and 4 studies of MMPs analyzed both Medicare and Medicaid spending (eTable 1 in Supplement 1).

**Table 2.** Summary of Findings, by Type of Integrated Care Plan (ICP)

Type	Studies assessing outcome, No.	Hypothesized ICP effect^a^	Principal findings		
			Support hypothesis	Contradict hypothesis	Null or mixed
**PACE**					
Spending					
Medicare spending	1	Reduction	0	0	1
Medicaid spending	3	Reduction	2	1	0
Utilization					
Long-term nursing home stays	4	Reduction	3	0	1
Hospital admissions, overall	2	Reduction	1	0	1
Skilled nursing facility use	0	Reduction	0	0	0
ED visits	2	Reduction	1	0	1
Outpatient visits (excluding ED)	0	Increase	0	0	0
HCBS	0	Increase	0	0	0
Coordination and quality of care					
Care coordination	0	Increase	0	0	0
Hospital readmissions	0	Reduction	0	0	0
Hospital admissions for ambulatory care-sensitive conditions	0	Reduction	0	0	0
Patient experience and health outcomes					
Patient satisfaction with care	0	Improvement	0	0	0
Mortality	3	Reduction	1	0	2
**MMPs**					
Spending					
Medicare spending	10	Reduction	0	8	2
Medicaid spending	4	Reduction	0	2	2
Utilization					
Long-term nursing home stays	8	Reduction	4	2	2
Hospital admissions, overall	10	Reduction	2	2	6
Skilled nursing facility use	9	Reduction	3	2	4
ED visits	10	Reduction	1	3	6
Outpatient visits (excluding ED)	9	Increase	4	0	5
HCBS	2	Increase	1	0	1
Coordination and quality of care					
Care coordination	8	Increase	1	0	7
Hospital readmissions	8	Reduction	2	2	4
Hospital admissions for ambulatory care-sensitive conditions	8	Reduction	1	1	6
Patient experience and health outcomes					
Patient satisfaction with care	1	Improvement	0	0	1
Mortality	0	Reduction	0	0	0
**FIDE-SNPs and similar managed care models**					
Spending					
Medicare spending	0	Reduction	0	0	0
Medicaid spending	0	Reduction	0	0	0
Utilization					
Long-term nursing home stays	5	Reduction	3	0	2
Hospital admissions, overall	5	Reduction	1	1	3
Skilled nursing facility use	2	Reduction	0	0	2
ED visits	5	Reduction	1	1	3
Outpatient visits (excluding ED)	3	Increase	2	0	1
HCBS	4	Increase	2	0	2
Coordination and quality of care					
Care coordination	1	Increase	0	0	1
Hospital readmissions	2	Reduction	0	0	2
Hospital admissions for ambulatory care−sensitive conditions	2	Reduction	1	0	1
Patient experience and health outcomes					
Patient satisfaction with care	1	Improvement	1	0	0
Mortality	2	Reduction	2	0	0

Abbreviations: ED, emergency department; FIDE-SNPs, Fully Integrated Dual Eligible Special Needs Plans; HCBS, home- and community-based services; MMPs, Medicare-Medicaid Plans; PACE, Programs of All-Inclusive Care for the Elderly.

^a^ Hypothesized effect reflects the difference in the outcome expected compared with nonintegrated coverage for dual-eligible beneficiaries.

### Health Service Utilization

Seventeen studies evaluated long-term nursing home stays (ie, stays exceeding 90-100 days), including 4 studies of PACE, 8 of MMPs, and 5 of FIDE-SNPs and other aligned models (Table 2). Three studies associated PACE with a lower likelihood of long-term nursing home placement.^12,22,36^ One study found that PACE enrollees had greater cognitive impairment on nursing home entry, suggesting that the program enabled beneficiaries to remain in the community longer before transitioning to a nursing home.^36^ Another study found PACE enrollees had a 2 to 4 percentage points (pp) lower risk of long-term nursing home stays in any 6-month interval compared to Medicaid enrollees in an HCBS waiver program, although it did not find a cumulative difference in long-term nursing home use. Using a broader comparison group of HCBS waiver and long-term nursing home-eligible enrollees, the study found lower 6-month and cumulative long-term nursing home use in PACE.^12^ Four studies found an association of MMPs with annual reductions in the probability of long-term nursing home stays of 0.5 to 4.2 pp (10.1% to 24.7% lower than baseline),^24,27,28,33^ while 2 associated MMPs with increases in nursing home stays and 2 reported null findings.^21,30^ Three studies found an association between FIDE-SNPs or other aligned programs and reductions in long-term nursing home use, 2 reported reductions of 16% to 68% relative to baseline, and 1 estimated that a 10-pp increase in aligned plan enrollment was associated with 0.3 fewer nursing home residents per month per 100 beneficiaries.^17,19,22^ Null findings for long-term nursing home use were reported in 1 study of PACE and 2 studies of FIDE-SNPs and other aligned models.^18,34,37^

Twelve studies evaluated outpatient care (9 of MMPs, 3 of FIDE-SNPs, and no studies of PACE). Increases in outpatient care were found in 4 studies of MMPs and 2 of FIDE-SNPs and other aligned models.^18,20,25,28,29,30^ Null findings were reported in 5 studies of MMPs and 1 study of an FIDE-SNP.^21,24,27,33,37,38^ No studies reported reductions in outpatient care.

HCBS use was assessed in 2 studies of MMPs and 4 studies of FIDE-SNPs and other aligned models. Two studies of FIDE-SNPs found that dual-eligible beneficiaries in these plans used substantially more HCBS than enrollees in nonintegrated plans.^22,37^ One study of Massachusetts’ MMP, which used a regression discontinuity design based on the age cutoff for program eligibility (which covered dual-eligible beneficiaries 64 years and younger) found that beneficiaries just under the program’s age eligibility threshold were 5.1% more likely to receive a health assessment to receive HCBS compared with individuals older than this threshold.^21^ However, a study of California’s MMP found no difference in enrollee-reported use of in-home support services compared to dual-eligible beneficiaries in California counties where the MMP was not offered.^38^ No studies of PACE evaluated HCBS use as an outcome, likely because supportive services in adult day health centers are embedded in this program’s design.

Studies of MMPs and FIDE-SNPs and other aligned models mostly reported inconsistent or null findings for hospital admissions, emergency department visits, and skilled nursing facility use.^17,18,19,20,21,27,28,29,30,33,34,37,38,39^ These outcomes were evaluated in few or no studies of PACE.

### Coordination and Quality of Care

A process-related care coordination measure, such as having a follow-up outpatient visit after a hospital stay, was reported in 9 studies (8 of MMPs, 1 of an FIDE-SNP, and none of PACE). All but 1 of these studies^24^ found no difference in care coordination associated with ICPs.^25,27,28,29,30,33,37,38^ All-cause 30-day hospital readmissions were evaluated in 10 studies (8 of MMPs and 2 of FIDE-SNPs or other aligned models), which reported varying findings (fewer readmissions in 2 studies of MMPs,^24,27^ increases in 2 studies of MMPs,^28,33^ and no difference in 4 studies of MMPs and 2 of FIDE-SNPs^20,21,25,29,30,40^). Studies mostly reported null findings regarding hospitalizations for ambulatory care-sensitive conditions.^21,24,25,29,30,33,37^

### Patient Experience

Only 2 studies evaluated patient-reported experiences with care. One study found few differences in care satisfaction between enrollees in California’s MMP compared with dual-eligible beneficiaries in California counties where the MMP was not offered.^38^ A study using national Consumer Assessment of Healthcare Providers and Systems data from 2015 to 2018 found that FIDE-SNP enrollees reported higher overall ratings of their health plan than dual-eligible beneficiaries in conventional MA plans and coordination-only D-SNPs. However, FIDE-SNPs received comparable or lower ratings in other care domains.^41^

### Health Outcomes

The most common health outcome studied was mortality (assessed in 3 studies of PACE and 2 of FIDE-SNPs and other aligned models). Two studies reported no difference in mortality between enrollees of PACE compared with nonintegrated arrangements,^22,34^ while 2 studies of FIDE-SNPs and 1 of PACE associated these programs with lower mortality.^12,17,22^ However, researchers cautioned that mortality differences could reflect selection bias rather than a causal effect of integrated care.^12,22^ For example, Ghosh et al^12^ estimated that cohorts of new PACE enrollees had lower mortality rates over 5 years (8-17 pp) than matched comparison cohorts of dual-eligible beneficiaries entering nursing homes or HCBS waiver programs. This difference narrowed to 2 to 5 pp when PACE enrollees were compared only to HCBS waiver enrollees. Due to the sensitivity of estimates to the comparison group, and the potential that matching did not fully account for confounders, the researchers concluded that selection bias was a concern.^12^ No studies evaluated patient-reported health.

### Subgroup Analyses

Fourteen studies evaluated beneficiaries with frailty or needing nursing home−level care,^12,19,22,24,25,27,28,29,30,33,34,35,36,37^ including all studies of PACE (reflective of the program’s eligibility criteria). Seven studies evaluated beneficiaries with a serious mental illness,^24,25,27,28,29,30,33^ and 2 studies evaluated people with cognitive impairment or an intellectual disability.^32,34^ Studies assessing these subgroups evaluated different outcomes, and there were often few subgroup-specific findings per outcome (eTable 3 in Supplement 1). Consequently, there was insufficient evidence to characterize the overall direction of findings for specific beneficiary subgroups.

### Study Design and Quality of Evidence

Fourteen studies used observational designs that compared dual-eligible beneficiaries in ICPs vs nonintegrated arrangements (all studies of PACE, 2 of MMPs, and 7 of FIDE-SNPs and other aligned models) (Table 1). Five observational studies used cross-sectional designs that compared enrollees in ICPs vs nonintegrated plans, either at a single point in time or pooled over several periods.^18,22,38,41^ Nine observational studies used longitudinal designs that compared changes in outcomes between cohorts of beneficiaries enrolled in ICPs vs nonintegrated plans.^12,17,20,34,35,36,37,39,40^ In both categories of observational studies, researchers used propensity score weighting, matching, or covariate adjustment to control for observed enrollee characteristics (eg, chronic conditions) that differed across plans. However, the quality of this evidence was rated as limited because the studies could not control for unmeasured confounders, such as differences in frailty, mobility, or social support.

Twelve studies—all but 1 assessing MMPs^19^—used quasi-experimental methods to mitigate bias from unmeasured confounders. Ten MMP studies used difference-in-differences designs that compared outcome changes among dual-eligible beneficiaries in counties where MMPs were implemented vs counties where these plans were unavailable.^24,25,26,27,28,29,30,31,32,33^ This approach leverages policy-driven changes in availability of MMPs to identify treatment effects, which can reduce bias because these changes are unlikely to be correlated with unmeasured person-level confounders.^16^ Another study used a regression discontinuity design to compare beneficiaries across the age eligibility cutoff (≤64 years) for Massachusetts’ MMP.^21^ These authors showed that beneficiary characteristics were balanced across this cutoff, suggesting that outcome differences across the cutoff were associated with OneCare eligibility. However, this approach identifies treatment effects close to the cutoff, which may not generalize to the broader population of dual-eligible beneficiaries. Among quasi-experimental studies, the quality of evidence was rated as moderate due to bias concerns and generalizability limitations.

## Discussion

This systematic review found considerable variability and gaps in evidence about the association of ICPs with health care quality, spending, utilization, and patient-reported and other health outcomes among dual-eligible beneficiaries. Six key themes emerged in this review. First, studies generally found reductions or delays in the onset of long-term nursing home care in PACE, FIDE-SNPs, and other aligned models, but evidence regarding long-term nursing home use was mixed in evaluations of MMPs. Second, several studies of MMPs and FIDE-SNPs or other aligned models found increased outpatient care and HCBS use, but other studies of these ICPs showed no difference. Third, across ICP categories, mostly null or inconsistent findings were reported for care coordination and hospitalizations. Fourth, most studies associated MMPs with higher Medicare spending, although few studies evaluated Medicaid spending, and no evaluations of FIDE-SNPs or similar aligned models evaluated spending, to our knowledge. Fifth, evidence was limited regarding patient satisfaction, beneficiary health, and outcomes in clinically important subpopulations of dual-eligible beneficiaries, such as those with serious mental illness. Sixth, studies often had limited ability to control for selection bias. Therefore, although the literature suggests that some ICPs are associated with outcomes aligned with policy goals (eg, reducing long-term nursing home care), it also highlights limitations of existing evidence that require urgent attention.

A notable limitation was that the availability of evidence varied across ICPs and was insufficient to evaluate outcomes for certain plan types. For example, patient satisfaction with care was evaluated in only 1 study of an MMP and 1 of FIDE-SNPs, spending was not evaluated in any study of FIDE-SNPs, and HCBS use was not analyzed in evaluations of PACE. However, this last omission may reflect that supportive services in adult day health centers are integral to PACE’s design, and thus, were not considered an outcome.

Furthermore, most studies relied on administrative data (eg, claims or encounter data), while few surveyed patients about their access to or experiences with care. This presents challenges for assessing whether utilization and spending changes reflected improved or worsened care. For example, it is not necessarily the case that better care management should lead to uniform increases or decreases in utilization and spending, and improved access to care could increase beneficiaries’ use of and spending on some services. Likewise, limited data on patient-reported outcomes (eg, getting help with activities of daily living or engagement with a care coordinator) made it difficult to assess whether ICPs addressed beneficiaries’ care needs.

Therefore, this review highlights several areas where research is needed to rigorously appraise the performance of ICPs. First, research is needed to evaluate combined Medicaid and Medicare spending. Second, assessment of patient-reported outcomes is critical for evaluating whether ICPs improve care in areas salient to dual-eligible beneficiaries, such as care coordination and quality of long-term care. Third, research is needed to assess outcomes in subgroups of dual-eligible beneficiaries with complex needs.

Fourth, our review highlights a need to address methodological limitations of the literature, most of which stem from the challenge of identifying appropriate comparison groups. Because enrollment in ICPs is voluntary, and beneficiaries may opt out of these plans if assigned to one, it is often difficult to identify a comparison group of beneficiaries that controls for selection bias. All evaluations of PACE and all but 1 study of FIDE-SNPs and aligned models used observational designs with propensity score weighting, matching, or covariate adjustment to account for observed enrollee characteristics across plan types. However, these designs are susceptible to bias from unmeasured differences between enrollees of ICPs and comparison plans. Some observational studies used longitudinal cohort designs that followed dual-eligible beneficiaries over time. Although longitudinal designs control for enrollee-level factors that remain constant over time, challenges arise if unobserved factors are correlated with outcome trends or mortality.^12,37^ Several studies used quasi-experimental designs to mitigate selection bias concerns; however, all but 1 of these studies evaluated MMPs. Further research that leverages policy-driven variation in ICP enrollment (eg, default enrollment into integrated plans) or randomized clinical designs could strengthen and broaden the evidence base on ICPs.

### Limitations

Other gaps in evidence limit the conclusions of this review. Because studies varied in populations and outcomes evaluated, we were unable to make head-to-head comparisons of the performance of different ICPs. Furthermore, studies provided little or no detail on contextual factors that could have influenced plan performance, such as plans’ care management practices or prior experiences with integrated care. Research assessing these factors and their relationship to outcomes could help policymakers identify and disseminate features of ICPs that may be associated with better care for dual-eligible beneficiaries.

## Conclusions

Findings from this systematic review highlight the variability in the association of ICPs with spending, utilization, and outcomes among dual-eligible Medicare beneficiaries, as well as gaps in the available evidence. Some studies associated some ICPs with reductions in long-term nursing home care, and several studies identified greater outpatient care use. However, evidence on spending—specifically on Medicaid—was limited for PACE and FIDE-SNPs, whereas MMPs were generally associated with higher Medicare spending. Evidence was mixed or insufficient to evaluate the association of ICPs with hospitalizations, care coordination, patient satisfaction, health, and outcomes among beneficiary subgroups in vulnerable situations. Research addressing these evidence gaps is urgently needed to guide integration policy for dual-eligible Medicare and Medicaid beneficiaries.

## Notes

Supplement 1. eMethods. eTable 1. Evaluations Examining Both Medicare and Medicaid Spending eTable 2. Subgroup Analyses eTable 3. Data Extracted from Individual Studies

Supplement 2. Data Sharing Statement.
