# Supplementary material for: Quality, Spending, Utilization, and Outcomes Among Dual-Eligible Medicare-Medicaid Beneficiaries in Integrated Care Programs: A Systematic Review
Source: JAMA Health Forum. 2024 Jul 19;5(7):e242187. doi: 10.1001/jamahealthforum.2024.2187 (PMC11259897; doi:10.1001/jamahealthforum.2024.2187)
Supplement: Supplement 1. — eMethods. eTable 1. Evaluations Examining Both Medicare and Medicaid Spending eTable 2. Subgroup Analyses eTable 3. Data Extracted from Individual Studies [file jamahealthforum-e242187-s001.pdf]

Supplemental Online Content

Roberts ET, Duggan C, Stein R, Jonnadula S, Johnston KJ, Figueroa JF. Quality, Spending, Utilization, and Outcomes Among Dual-Eligible Medicare-Medicaid Beneficiaries in Integrated Care Programs: A Systematic Review. *JAMA Health Forum.* 2024;5(7):e242187. doi:10.1001/jamahealthforum.2024.2187

eMethods.

eTable 1. Evaluations Examining Both Medicare and Medicaid Spending

eTable 2. Subgroup Analyses

eTable 3. Data Extracted from Individual Studies

This supplemental material has been provided by the authors to give readers additional information about their work.

© 2024 Roberts et al. *JAMA Health Forum.*

eMethods: Methodology

This systematic review was conducted in accordance with PRISMA methodology for systematic reviews.(1)

This review was designed according to the following PECOS framework:

Population: Dual eligible beneficiaries (enrolled in Medicare and full Medicaid), including both community-dwelling and institutionalized populations.
Exposures: Enrollment in an ICP (defined as a Program of All-Inclusive Care for the Elderly [PACE], a capitated Medicare-Medicaid Plan [MMP], Highly Integrated D-SNPs [HIDE-SNPs], Fully Integrated D-SNPs [FIDE-SNPs], or similar state-based model).[1]
Comparators: Enrollment in nonintegrated Medicare and Medicaid products (e.g., where Medicare and Medicaid benefits and spending are managed by separate entities). This can include: 1) coordination-only D-SNPs, 2) Medicare Advantage plans that are not financially integrated D-SNPs, or 3) fee-for-service Medicare. Comparator groups may have fee-for-service Medicaid or Medicaid managed care (including a Medicaid HCBS waiver program) provided that it is not financially integrated with Medicare.
Outcomes: 1) utilization of care (e.g., per-capita use of outpatient, acute, long-term, and post-acute care); 2) quality of care (e.g., care coordination); 3) patient experience with care (e.g., satisfaction with care) and health outcomes (e.g., functional status and mortality); and 4) Medicare and Medicaid spending. We also assessed whether studies reported the effects of ICPs on these outcomes for subgroups of beneficiaries (e.g., those with a serious mental illness or needing long-term services and supports).
Setting: US population.

To capture all studies which may fulfill the above criteria, we searched PubMed and Google Scholar as well as government websites, including websites for the Centers for Medicare and Medicaid Services (CMS), Health and Human Services/Assistant Secretary for Planning and Evaluation (HHS/ASPE), the Medicaid and CHIP Payment and Access Commission (MACPAC), and the Medicare Payment Advisory Commission (MedPAC). We searched Google Scholar to identify relevant literature beyond academic journals, including government reports and program evaluations, which are not indexed on PubMed. The above-referenced government websites were also searched directly to ensure that we included all relevant government reports (even if not indexed on Google Scholar).

Syntax for our PubMed and Google Scholar queries is reported below. We developed this syntax iteratively by using it to search for studies identified through our prior work and verified that the results returned by these queries included previously identified studies of integrated care plans.

PubMed Query:
(("integrat*"[tiab]) AND (("dual#eligible*"[tiab]) OR ("dually eligible"[tiab]))) **OR** (("align*"[tiab]) AND (("dual#eligible*"[tiab]) OR ("dually eligible"[tiab]))) **OR** (“dual eligible special needs plan"[tiab:~0]) **OR** (“dual eligible special needs plans"[tiab:~0]) **OR** ((“D-SNP*”[tiab]) AND (("dual#eligible"[tiab]) OR ("dually eligible"[tiab]))) **OR** ("Program of All-Inclusive Care for the Elderly"[tiab:~0]) **OR** ("Program for All-Inclusive Care for the Elderly"[tiab:~0]) **OR** (("PACE"[tiab]) AND (("dual#eligible*"[tiab]) OR ("dually

© 2024 Roberts et al. *JAMA Health Forum.*

eligible"[tiab]))) **OR** ("financial alignment initiative"[tiab:~0]) **OR** ("Medicare-Medicaid Plan"[tiab:~0]) **OR** ((“MMP” [tiab]) AND (("dual#eligible*"[tiab]) OR ("dually eligible"[tiab]))) **OR** ("Minnesota senior health options program"[tiab:~0]) **OR** ("Massachusetts Senior Care Options"[tiab:~0])

Google Scholar Queries:
Five separate searches restricted to publish date 2010-2023 (due to character limits for Google Scholar searches as well as restrictions on the number of query results that could be bulk downloaded). Citation records and patents were excluded from the results. Searches run through Publish or Perish.

1. "dual-eligible special needs plans" OR "dual special needs plans" OR “dual-eligible Medicare Advantage special needs plans”
2. ("dual eligible*" OR "dually eligible") AND ("Program of All-Inclusive Care for the Elderly" OR "Program for All-Inclusive Care for the Elderly")
3. "financial alignment initiative"
4. "Medicare-Medicaid Plan" OR "Medicare-Medicaid Plans"
5. "Minnesota senior health options program" OR "Massachusetts Senior Care Options"

Inclusion/Exclusion:
After searching, we used additional inclusion/exclusion criteria beyond the PECOS framework to screen studies. First, we intentionally excluded small quantitative studies and qualitative studies consisting of interviews or focus groups, as these studies do not provide robust outcomes of interest to quantitatively compare across studies. Second, we excluded studies that summarized findings from prior research but did not present original empirical evidence. Third, we excluded conference publications, abstracts (not accompanied by a full study), books, book chapters, dissertations, and reports/studies from commercial entities (such as health plans) that were not published in a peer-reviewed journal or on a government website. Fourth, studies published before 2010 were excluded, as ICPs have changed significantly since then, and thus, those findings are less relevant to current programs. Fifth, we did not include studies reporting interim or preliminary findings if we identified a more recent study providing updated or final findings. (For example, we only cited the most recent evaluations of Medicare-Medicaid Plans at the time of this review.) Sixth, we excluded evaluations of Financial Alignment Initiative demonstrations in Washington and Colorado because those demonstrations used a fee-for-service model, whereas most proposals to expand ICPs focus on capitated, managed care models.

To be considered for **inclusion**, a study must:

1. Compare at least 1 outcome (e.g., utilization, quality, patient experience or health outcome, or spending) among:
    a. Dual eligibles enrolled in an ICP to those enrolled in a non-ICP, <u>*or*</u>
    b. Dual eligibles ‘exposed’ to the implementation of an ICP (e.g., due to introduction of a new model) to ‘unexposed’ dual eligibles. This covers evaluations of MMPs, which used intention-to-treat designs to compare dual eligibles in regions newly implementing MMPs to those in control regions.

We note that descriptions of ICPs vary in the literature. In addition to the ICP models listed above, we will consider programs where dual eligibles beneficiaries were

© 2024 Roberts et al. *JAMA Health Forum.*

enrolled in 'aligned' Medicare and Medicaid managed care plans operated by the same insurers to be integrated. We take such descriptions to functionally represent integration, unless it is clear from a detailed read of the study that no financial integration occurred.

2. Have been published in a peer-reviewed journal or be a program evaluation (e.g., an evaluation prepared for CMMI, ASPE, or a state) published on a public government website. Conference publications, abstracts (not accompanied by a full study), books, book chapters, theses, and dissertations will be excluded.
3. Report quantitative findings for a sample consisting of at least 100 study subjects.
4. Be published or accepted for publication in English between January 1, 2010 and October 1, 2023 (Google Scholar Query) and through November 1, 2023 (PubMed and government websites). We incorporated findings from 3 MMP evaluations updated in December 2023.

Exclusion criteria:

1. Studies without a contemporaneous comparison group, such as a nonintegrated care program or populations that are ineligible for an ICP, will be excluded.
2. Studies that only include coordination-only D-SNPs will be excluded, as coordination-only D-SNPs do not manage Medicaid spending for dual eligible enrollees.
3. Studies that do not primarily focus on dual eligibles as the target population of reforms (e.g., that only consider dual eligibles as a subpopulation of a broader reform) will be excluded.
4. Publications that are not empirical evaluations of an ICP will be excluded (e.g., publications that are editorials, commentaries, etc., will be excluded).

We note that, for federally funded evaluations of MMPs, we only reported findings from empirical analyses that compared dual-eligible individuals eligible for an MMP to those ineligible (e.g., because of geographic variation in MMP implementation). Although these evaluations also reported HEDIS and patient-reported experience measures for MMPs and other Medicare Advantage plans, we did not summarize findings because they are based on comparisons of MMPs, which exclusively serve dual-eligible beneficiaries, to other Medicare Advantage plans, which may serve both dual-eligible and non-dual-eligible beneficiaries.

1. Page MJ, McKenzie JE, Bossuyt PM, Boutron I, Hoffmann TC, Mulrow CD, et al. The PRISMA 2020 statement: an updated guideline for reporting systematic reviews. BMJ. 2021 Mar 29;372:n71.

© 2024 Roberts et al. *JAMA Health Forum.*

eTable 1: Evaluations Examining Both Medicare and Medicaid Spending

Study	Finding for Medicare Spending	Finding for Medicaid Spending
**PACE Program:** Ghosh et al., Effects of PACE on Costs, Nursing Home Admissions, and Mortality 2006 - 2011, 2014	No change	Increased
**MMP Evaluation:** Snow et al., New York Fully Integrated Duals Advantage for Individuals with Intellectual and Developmental Disabilities: Preliminary Third Evaluation Report, October 2023	No change	No change
**MMP Evaluation:** Gattine et al., Financial Alignment Initiative Massachusetts One Care: Preliminary Fifth Evaluation Report	Increased	Increased
**MMP Evaluation:** Griffin et al., Texas Dual Eligible Integrated Care Demonstration Preliminary Third Evaluation Report, December 2023	No change	No change
**MMP Evaluation:** Khatutsky et al, California Cal MediConnect Preliminary Third Evaluation Report, April 2023	Increased	Increased

© 2024 Roberts et al. *JAMA Health Forum.*

eTable 2: Subgroup Analyses

	Number of Studies examining outcome	Hypothesized effect of integrated program [a]	Principal findings		
			Finding supports hypothesized effect of integrated care program	Finding contradicts hypothesized effect of integrated care program	Null or mixed finding
**Frail or needing nursing home-level care (14 studies)**					
**Spending**					
Medicare spending	1	Reduction	0	0	1
Medicaid spending	3	Reduction	2	1	0
**Utilization**					
Long-term nursing home stays	5	Reduction	3	2	0
Hospital admissions, overall	11	Reduction	3	2	6
Skilled nursing facility use	8	Reduction	0	3	5
Emergency department (ED) visits	11	Reduction	3	2	6
Outpatient visits (excluding ED visits)	8	Increase	1	0	7
Home and community-based services (HCBS)	2	Increase	1	1	0
**Management and Quality of Care**					
Care coordination	8	Increase	0	0	8
Hospital readmissions	7	Reduction	1	1	5
Hospital admissions for ambulatory care-sensitive conditions (ACSCs)	8	Reduction	1	2	5
**Patient experience and health outcomes**					
Patient satisfaction with care	0	Improvement	0	0	0

© 2024 Roberts et al. *JAMA Health Forum.*

Mortality	3	Reduction	1	0	2
**Serious and persistent mental illness (7 studies)**					
**Spending**					
Medicare spending	0	Reduction	0	0	0
Medicaid spending	0	Reduction	0	0	0
**Utilization**					
Long-term nursing home stays	0	Reduction	0	0	0
Hospital admissions, overall	7	Reduction	3	0	4
Skilled nursing facility use	7	Reduction	2	2	3
ED visits	7	Reduction	0	2	5
Outpatient visits (excluding ED visits)	7	Increase	3	0	4
HCBS	0	Increase	0	0	0
**Coordination and Quality of Care**					
Care coordination	0	Increase	0	0	0
Hospital readmissions	7	Reduction	1	1	5
Hospital admissions for ACSCs	7	Reduction	1	0	6
**Patient experience and health outcomes**					
Patient satisfaction with care	0	Improvement	0	0	0
Mortality	0	Reduction	0	0	0
**Intellectual and Developmental Disabilities (1 study)**					
**Spending**					
Medicare spending	1	Reduction	0	0	1
Medicaid spending	1	Reduction	0	0	1
**Utilization**					
Long-term nursing home stays	0	Reduction	0	0	0
Hospital admissions, overall	0	Reduction	0	0	0

© 2024 Roberts et al. *JAMA Health Forum.*

Skilled nursing facility use	0	Reduction	0	0	0
ED visits	0	Reduction	0	0	0
Outpatient visits (excluding ED visits)	0	Increase	0	0	0
HCBS	0	Increase	0	0	0
**Coordination and Quality of Care**					
Care coordination	0	Increase	0	0	0
Hospital readmissions	0	Reduction	0	0	0
Hospital admissions for ACSCs	0	Reduction	0	0	0
**Patient experience and health outcomes**					
Patient satisfaction with care	0	Improvement	0	0	0
Mortality	0	Reduction	0	0	0
**Greater Cognitive Needs (1 study)**					
**Spending**					
Medicare spending	0	Reduction	0	0	0
Medicaid spending	1	Reduction	1	0	0
**Utilization**					
Long-term nursing home stays	0	Reduction	0	0	0
Hospital admissions, overall	0	Reduction	0	0	0
Skilled nursing facility use	0	Reduction	0	0	0
ED visits	0	Reduction	0	0	0
Outpatient visits (excluding ED visits)	0	Increase	0	0	0
HCBS	0	Increase	0	0	0
**Coordination and Quality of Care**					
Care coordination	0	Increase	0	0	0
Hospital readmissions	0	Reduction	0	0	0
Hospital admissions for ACSCs	0	Reduction	0	0	0

© 2024 Roberts et al. *JAMA Health Forum.*

**Patient experience and health outcomes**					
Patient satisfaction with care	0	Improvement	0	0	0
Mortality	0	Reduction	0	0	0

Source: Authors' analysis of included studies.
Notes: [a] Hypothesized effect reflects the difference in the outcome expected relative to nonintegrated coverage for dual-eligible beneficiaries.

© 2024 Roberts et al. *JAMA Health Forum.*

eTable 3: Data Extracted from Individual Studies
This section provides detailed information about data abstracted from each study included in this review.

General Study Information Part 1

Study Author, Study Title, and Year	Type of ICP Evaluated	Number of Participants for the ICP	Enrolled or Eligible Participants	Notes on Participant Number
**Chapin et al, Program of All-inclusive Care for the Elderly (PACE) Medicaid Cost-Benefit Study, 2013**	PACE	136	Enrolled	The PACE group were those who remained in PACE for entire time period (and excluded those who switched) while the control groups kept switchers (see page 15 for details)
**Segelman et al., Transitioning from Community-Based to Institutional Long-term Care: Comparing 1915(c) Waiver and PACE Enrollee 2017**	PACE	4733	Enrolled	New PACE enrollees in 12 states were defined as those who were enrolled in given month but not in the prior 3 months (only first occurrence of enrollment in study period counted). Enrollees who disenrolled from PACE more than 90 days prior to NH entry were excluded from study.
**Ghosh et al., Effects of PACE on Costs, Nursing Home Admissions, and Mortality 2006 - 2011, 2014**	PACE	3725	Enrolled	
**Wieland et al, Does Medicaid Pay More to a Program of All-Inclusive Care for the Elderly (PACE) Than for Fee-for-Service Long-term Care?, 2013**	PACE	948	Enrolled	
**Feng et al., Comparing Outcomes for Dual Eligible Beneficiaries in Integrated Care: Final Report, 2021**	PACE	25665	Enrolled	
**Griffin et al., MyCare Ohio: Third Evaluation Report, October 2023**	MMP	141966	Eligible	In 2021, 58% of eligible individuals were enrolled in the program.

© 2024 Roberts et al. *JAMA Health Forum.*

Study Author, Study Title, and Year	Type of ICP Evaluated	Number of Participants for the ICP	Enrolled or Eligible Participants	Notes on Participant Number
**Snow et al. Financial Alignment Initiative New York Fully Integrated Duals Advantage for Individuals with Intellectual and Developmental Disabilities: Preliminary Third Evaluation Report. 2023.**	MMP	22,488	Eligible	Although approximately 22,000 individuals were eligible to enroll in this model, only 7.5% were enrolled in 2021. Evaluators cited lack of participation by major hospital systems to be a major factor influencing eligible beneficiaries' choices to not enroll.<br><br>Note that New York's demonstration, called "Fully Integrated Duals Advantage for Individuals with Intellectual and Developmental Disabilities" (FIDA-IID), exclusively served dual-eligible beneficiaries with intellectual and developmental disabilities (IDD) who were age 21 and older and resided in demonstration counties.
**Holladay et al., Illinois Medicare-Medicaid Alignment Initiative: Third Evaluation Report, November 2022**	MMP	263128	Eligible	Demonstration was expanded statewide in 2021, increasing the number of eligible individuals from 169,000 to 263,000 from 2020 to 2021. In 2021, 36% of eligible individuals were enrolled in the program. However, this evaluation only includes quantitative outcomes through 2019.
**Chepaitis et al., Virginia Commonwealth Coordinated Care Evaluation Report, Spring 2021**	MMP	57937	Eligible	37% of eligible individuals were enrolled in the Virginia Coordinated Care MMP in 2017, the program's final year.

© 2024 Roberts et al. *JAMA Health Forum.*

**Study Author, Study Title, and Year**	**Type of ICP Evaluated**	**Number of Participants for the ICP**	**Enrolled or Eligible Participants**	**Notes on Participant Number**
**Howard et al., South Carolina Healthy Connections Prime Third Evaluation Report, December 2023**	MMP	25,410	Eligible	59% of eligible individuals were enrolled in the program in December 2021.
**Gattine et al., Financial Alignment Initiative Massachusetts One Care: Preliminary Fifth Evaluation Report**	MMP	118443	Eligible	27% of eligible individuals enrolled in the program in 2019.
**Holladay et al., Financial Alignment Initiative Michigan MI Health Link Second Evaluation Report, March 2022**	MMP	109548	Eligible	37% of eligible individuals were enrolled in the program in 2020.
**Gattine et al. Rhode Island Integrated Care Initiative: Third Evaluation Report, December 2023**	MMP	37,126	Eligible	40% of eligible individuals enrolled in demonstration in 2019. Enrollment reached 13,000 in December 2021 (35% of the eligible population).
**Griffin et al., Texas Dual Eligible Integrated Care Demonstration: Preliminary Third Evaluation Report, December 2023**	MMP	157,348	Eligible	23.4% of eligible individuals enrolled in demonstration in 2020.
**Khatutsky et al, California Cal MediConnect Preliminary Third Evaluation Report, April 2023**	MMP	479461	Eligible	Approximately 24% of eligible individuals were enrolled in the Cal MediConnect demonstration in 2021.

© 2024 Roberts et al. *JAMA Health Forum.*

Study Author, Study Title, and Year	Type of ICP Evaluated	Number of Participants for the ICP	Enrolled or Eligible Participants	Notes on Participant Number
**Caswell et al., Do Integrated Care Models for Dual Medicare-Medicaid Enrollees Work? Evidence from Massachusetts' One Care Financial Alignment Demonstration (In press, 2023)**	MMP	16680	Eligible	This is an intention-to-treat regression discontinuity design that compares individuals just below the age eligibility ceiling (<65 years) for OneCare vs. those just above this eligibility ceiling at baseline. Of those <65 years at the demonstration start, approximately 20% were ever enrolled in OneCare during the study window.<br><br>Note: Eligibility is measured at the start of the demonstration (2013) although outcomes are measured for the period 2016-2018 due to data availability constraints.
**Chen et al, Early evidence from South Carolina's Medicare-Medicaid dual-eligible financial alignment initiative: an observational study to understand who enrolled, and whether the program improved health, 2018**	MMP	13370	Enrollees	Study estimates changes among program enrollees.
**Graham et al., Beneficiaries Respond To California's Program to Integrate Medicare, Medicaid, And Long-Term Services, 2018**	MMP	488	Enrolled	488 out of 744 CMC (Cal MediConnect) survey respondents (65% response rate). Total CMC enrollees by 2017 was ~120,000.
**Meyers et al., Medicare and Medicaid Dual-Eligible Special Needs Plan Enrollment and Beneficiary-Reported Experiences with Care, 2023**	FIDE-SNP	10565	Enrolled	MA-CAHPS data of full-benefit duals in FIDEs (43% response rate)

© 2024 Roberts et al. *JAMA Health Forum.*

Study Author, Study Title, and Year	Type of ICP Evaluated	Number of Participants for the ICP	Enrolled or Eligible Participants	Notes on Participant Number
**Roberts et al., Changes in care associated with integrating Medicare and Medicaid for dual eligible individuals, In Press 2023**	FIDE-SNP	7967	Enrolled	Study compared two cohorts: 1) an 'integration cohort' of D-SNP enrollees in Pennsylvania who joined a companion Medicaid managed care plan following a Pennsylvania reform mandating Medicaid managed care (leading to integration), and 2) a 'comparison cohort' of dual-eligible beneficiaries in Pennsylvania who remained enrolled in fee-for-service Medicare before and after the introduction of Medicaid managed care. Thus, coverage nonintegrated for the comparison cohort.
**Keohane et al., Aligning Medicaid and Medicare Advantage Managed Care Plans for Dual-Eligible Beneficiaries, 2021**	Other Integrated Model	129,731	Eligible (residing in counties in the study)	Analysis at the person-month for full-benefit duals in aligned D-SNP (same insurer for Medicaid and Medicare D-SNP), non-aligned D-SNP, traditional Medicare, and non-DSNP MA plan. Design is intention to treat
**Feng et al., Comparing Outcomes for Dual Eligible Beneficiaries in Integrated Care: Final Report, 2021**	FIDE-SNP	89,949	Enrolled	
**Kim et al, Comparing Care for Dual-Eligibles Across Coverage Models: Empirical Evidence from Oregon, 2019**	HIDE-SNP	34% of 50,941	Enrolled	Number of enrollees reported for the first quarter of 2011.

© 2024 Roberts et al. *JAMA Health Forum.*

Study Author, Study Title, and Year	Type of ICP Evaluated	Number of Participants for the ICP	Enrolled or Eligible Participants	Notes on Participant Number
**Anderson et al, Effects of Integrating Care for Medicare-Medicaid Dually Eligible Seniors in Minnesota, 2020**	Other Integrated Model	99761	Enrolled	Sample was limited to those consistently enrolled in MHSO (the ICP) or MSC+ within a full year across the 3-year period. On average, 12.8% of those enrolled in MSC+ at the beginning of the year switched to MSHO by the end of the year while less than 0.4% of MSHO enrolled switched back to MSC+ during a year.
**Jung et al, Integrated Medicare and Medicaid Managed Care and Rehospitalization of Dual Eligibles, 2015**	Other Integrated Model	1,090	Enrolled	
**Jen Associates, Massachusetts Senior Care Option 2005-2010 Impact on Enrollees: Nursing Home Entry Utilization August 14, 2013**	Other Integrated Model	12,064	Enrolled	For nursing home and survival analysis through 2010, there were 10,335 SCO enrollees and 29,169 controls in 2004-2009, excluding those with pre-index nursing home residence included.

© 2024 Roberts et al. *JAMA Health Forum.*

General Study Information Part 2

Study Author, Study Title, and Year	Scope/ representativeness of the ICP	Primary Data Sources	Characteristics of Dual Eligibles Studied
**Chapin et al, Program of All-inclusive Care for the Elderly (PACE) Medicaid Cost-Benefit Study, 2013**	Single state	Administrative data from the Medicaid Management Information Systems (MMIS) database and the Level of Care Threshold (LOC) score (which is based on a functional assessment designed to determine whether adults are eligible for NF care. Years: 2006 to 2011.	Adults 65y and older who were enrolled in LTC for at least 90 days that began between July 2006 to Dec 2011 (either in PACE or in 2 other options in the 8 PACE counties, including HCBS/Frail Elderly waiver and those in nursing facilities). Patients received no more than 60 days of service in one of the other Medicaid programs in the last 6 months prior to their first long-term stay.
**Segelman et al., Transitioning from Community-Based to Institutional Long-term Care: Comparing 1915(c) Waiver and PACE Enrollee 2017**	Multi-state but not national	MAX PS and MDS from 2005 to 2009.	Newly enrolled PACE enrollees in 12 states between 04/2005 to 12/2007 vs. those enrolled in 1915(c) aged or aged and disabled waiver programs (as controls) and followed through 2009; everyone was aged 55 years or older (to match PACE eligibility).
**Ghosh et al., Effects of PACE on Costs, Nursing Home Admissions, and Mortality 2006 - 2011, 2014**	Multi-state but not national	Medicare and Medicaid claims and encounter data (including Part D, MBSF, and MDS) from 2006-2011	Limited to beneficiaries who entered PACE between July 2006-December 2008, were at least 66 years old, were enrolled in Medicaid within a month of PACE enrollment, were enrolled in FFS Medicare for at least 1 month in the year prior to PACE enrollment (with non-missing utilization data) and had at least 1 month of enrollment during the follow-up period.
**Wieland et al, Does Medicaid Pay More to a Program of All-Inclusive**	Single state	South Carolina state Medicaid LLTC admission records and	Duals ages 55+ in South Carolina entering LTC from the community

© 2024 Roberts et al. *JAMA Health Forum.*

Study Author, Study Title, and Year	Scope/ representativeness of the ICP	Primary Data Sources	Characteristics of Dual Eligibles Studied
**Care for the Elderly (PACE) Than for Fee-for-Service Long-term Care?, 2013**		assessment data from 1994-2005	between 1994 and 2005. All beneficiaries were within the SC PACE two-county catchment area and qualified to enter any of the three cohorts (PACE, NH, or aged/disabled waiver).
**Feng et al., Comparing Outcomes for Dual Eligible Beneficiaries in Integrated Care: Final Report, 2021**	National	MA encounter data from CY 2015 with a four-year runout period through 2019 (to ensure data completeness) and some baseline 2014 Medicare risk adjustment data.	MA full-benefit duals who were consistently enrolled in PACE for all months they were alive and Medicare-eligible in 2015 and had 2014 baseline risk adjustment data.
**Griffin et al., MyCare Ohio: Third Evaluation Report, October 2023**	Single state	Administrative data, including Medicare claims and encounter data, Medicaid encounter data, nursing home Minimum Data Set, and other administrative data sources. Years for most data sources: 2012-2020.	Dual eligible beneficiaries ages 18 and older in 29 MyCare Ohio demonstration counties (excluding those enrolled in PACE). Some dual eligibles (e.g., those with Intellectual and Developmental Disabilities receiving waiver services) were excluded.

© 2024 Roberts et al. *JAMA Health Forum.*

Study Author, Study Title, and Year	Scope/ representativeness of the ICP	Primary Data Sources	Characteristics of Dual Eligibles Studied
**Snow et al. Financial Alignment Initiative New York Fully Integrated Duals Advantage for Individuals with Intellectual and Developmental Disabilities: Preliminary Third Evaluation Report. 2023.**	Single state	Cost data from Medicare Part A and Part B claims; Medicare Advantage and Part D Inquiry System (MARx) data; Medicaid Analytic Extract and Transformed Statistical Information System Analytic Files, 2014-2020. Utilization data were not available for this analysis.	Dual-eligible beneficiaries age 21 and older with intellectual and developmental disabilities (IDD), including those residing in Intermediate Care Facilities and the community. Demonstration was implemented in nine New York counties, including New York City and surrounding metropolitan counties.
**Holladay et al., Illinois Medicare-Medicaid Alignment Initiative: Third Evaluation Report, November 2022**	Single state	Medicare claims and encounter data, Medicaid encounter data, Minimum Data Set, and other administrative data sources. Years: 2012-2019. (Note: most outcomes are only examined through 2018, a ~5-year follow-up from the start date of the program.)	Dual eligible beneficiaries ages 21 and older in Illinois MMP in 21 counties in Greater Chicago (6 counties) and Central Illinois (15 counties). Residents of intermediate care facilities at baseline were ineligible.
**Chepaitis et al., Virginia Commonwealth Coordinated Care Evaluation Report, Spring 2021**	Single state	Administrative data, including Medicare claims and encounter data, Medicaid encounter data, nursing home Minimum Data Set, and other administrative data sources.	Dual eligible beneficiaries aged 21 or older who resided in the program's 114 demonstration counties (67 urban counties and 47 rural counties). Enrollees of PACE and certain waiver programs at baseline were ineligible.

© 2024 Roberts et al. *JAMA Health Forum.*

Study Author, Study Title, and Year	Scope/ representativeness of the ICP	Primary Data Sources	Characteristics of Dual Eligibles Studied
		Years: 2012-2017. Note: Medicaid claims and spending data were not available.	
**Howard et al., South Carolina Healthy Connections Prime Third Evaluation Report, December 2023**	Single state	Administrative data, including Medicare claims and encounter data, Medicaid encounter data, nursing home Minimum Data Set, and other administrative data sources. Years for most data sources: 2013-2020.	Dual eligible beneficiaries aged 65 or older who resided in the program's 43 demonstration counties (23 in metropolitan areas and 20 in non-metropolitan areas). Dual eligible beneficiaries younger than 65 years, residents of nursing facilities at the time of enrollment, and enrollees in PACE programs were ineligible.
**Gattine et al., Financial Alignment Initiative Massachusetts One Care: Preliminary Fifth Evaluation Report**	Single state	Administrative data, including Medicare claims and encounter data, Medicaid encounter data, nursing home Minimum Data Set, and other administrative data sources. Years for most data sources: 2011-2019.	Dual eligible beneficiaries ages 21-64 at the time of enrollment who resided in demonstration counties (12 counties in Massachusetts). Enrollees of PACE and residents of intermediate care facilities were ineligible.
**Holladay et al., Financial Alignment Initiative Michigan MI Health Link Second Evaluation Report, March 2022**	Single state	Administrative data, including Medicare claims and encounter data, Medicaid encounter data, nursing home Minimum Data Set, and other administrative data sources. Years for most data sources: 2013-2018.	Dual eligible beneficiaries ages 21+ living in Michigan counties participating in the demonstration (Detroit metro, SW Michigan, and upper peninsula). The demonstration areas included 42 counties—25 counties that are part of six MSAs and 17 non-metro counties. Enrollees of PACE or any other shared savings program were ineligible.

© 2024 Roberts et al. *JAMA Health Forum.*

**Study Author, Study Title, and Year**	**Scope/ representativeness of the ICP**	**Primary Data Sources**	**Characteristics of Dual Eligibles Studied**
**Gattine et al. Rhode Island Integrated Care Initiative: Combined Third Evaluation Report, December 2023**	Single state	Administrative data, including Medicare claims and encounter data, Medicaid encounter data, nursing home Minimum Data Set, and other administrative data sources. Years for most data sources: 2014-2020.	Dual eligible beneficiaries ages 21 and older. Residents of certain institutional care settings at baseline were ineligible.
**Griffin et al., Texas Dual Eligible Integrated Care Demonstration: Preliminary Third Evaluation Report, December 2023**	Single state	Medicare claims and encounter data, Medicaid encounter data, Minimum Data Set, and other administrative data sources. Years for most data sources: 2013-2020.	Dual eligible beneficiaries ages 21 and older in 6 metropolitan counties in Texas. Enrollees of PACE, intermediate care facilities, and certain Medicaid waiver programs were ineligible.
**Khatutsky et al, California Cal MediConnect Preliminary Third Evaluation Report, April 2023**	Single state	Medicare and Medicaid spending data (including capitation rates) from 2012-2019.<br><br>Report does not examine program impacts on service utilization because encounter data for California's MMP (needed to measure utilization in the integrated plan) were deemed incomplete	Dual eligible beneficiaries ages 21 and older in 7 metro counties (Los Angeles, Orange, Riverside, San Bernardino, San Diego, San Mateo, and Santa Clara). Enrollees of PACE and certain Medicaid waiver programs were ineligible.

© 2024 Roberts et al. *JAMA Health Forum.*

**Study Author, Study Title, and Year**	**Scope/ representativeness of the ICP**	**Primary Data Sources**	**Characteristics of Dual Eligibles Studied**
**Caswell et al., Do Integrated Care Models for Dual Medicare-Medicaid Enrollees Work? Evidence from Massachusetts' One Care Financial Alignment Demonstration (In press, 2023)**	Single state	Medicare and Medicaid administrative data for period 2016-2018	Dual eligible individuals just below the age eligibility ceiling for One Care (<65 years).
**Chen et al, Early evidence from South Carolina's Medicare-Medicaid dual-eligible financial alignment initiative: an observational study to understand who enrolled, and whether the program improved health, 2018**	Single state	Medicare and Medicaid administrative data from 2011-2016	Dual eligible beneficiaries in South Carolina who were eligible for Healthy Connections Prime (South Carolina's MMP)
**Graham et al., Beneficiaries Respond To California's Program to Integrate Medicare, Medicaid, And Long-Term Services, 2018**	Single state	Survey data of CMC members (random sample) in 2016 (first survey) and 2017 (second survey of same sample). Years 2016-2017. The initial survey was in Jan 2016, at which point CMC enrollees had been in program for 6 to 22 months).	Full benefit duals age 21+ who participated in the CMC demonstration across 7 counties in CA. Of note, duals in CMC were passively enrolled but could opt out (of which approximately 50% of those eligible opted out -- main reason given was to keep seeing their providers; concerns of not covering specific services/benefits they needed; being content with FFS Medicare; or found CMC too confusing/complicated). Beneficiaries had to have been enrolled in Medi-Cal for at least 6 months before transitioning to CMC.
**Meyers et al., Medicare and Medicaid Dual-Eligible Special Needs Plan Enrollment and**	National	MA-CAHPS data from 2015-2018	Excluded duals with more than 100 days of nursing home care in the survey year

© 2024 Roberts et al. *JAMA Health Forum.*

Study Author, Study Title, and Year	Scope/ representativeness of the ICP	Primary Data Sources	Characteristics of Dual Eligibles Studied
**Beneficiary-Reported Experiences with Care, 2023**			and in institutional and chronic condition SNPs
**Roberts et al., Changes in care associated with integrating Medicare and Medicaid for dual eligible individuals, In Press 2023**	Single state	D-SNP and Medicaid managed care administrative data from UPMC Health Plan, fee-for-service Medicare data, and Pennsylvania Medicaid program data (pre-Medicaid managed care) for the period 2015-2020.	Dual-eligible beneficiaries ages 21 and older who 1) were enrolled in a D-SNP operated by UPMC Health Plan at baseline (2015-2017), 2) enrolled in a companion Medicaid managed care plan operated by UPMC following a 2018 state reform mandating Medicaid managed care, and 3) who remained enrolled in these companion Medicare and Medicaid managed care plans, while alive, in the post-period (2018-2020).
**Keohane et al., Aligning Medicaid and Medicare Advantage Managed Care Plans for Dual-Eligible Beneficiaries, 2021**	Single state	TennCare eligibility files for Medicaid managed care plan coverage and the MMBSF for TM or MA participation. Medicaid plan encounter data was from TennCare for nursing home and HBCS services. TN hospital discharge data for IP and ED visits. Medicare Part D for prescription drug use.	Full duals for at least 1mo with linkable Medicaid and Medicare data (excluded 7.7% of duals <65 and 3.2% of duals 65+ because data could not be linked) plus other exclusions for missing plan identifiers, multiple beneficiary identifiers for Medicare to one Medicaid plan, out of state residence.
**Feng et al., Comparing Outcomes for Dual Eligible Beneficiaries in Integrated Care: Final Report, 2021**	National	MA encounter data from CY 2015 with a four-year runout period through 2019 (to ensure data completeness) and some baseline 2014	MA full-benefit duals who were consistently enrolled in a FIDE-SNP for all months they were alive and Medicare-eligible in 2015 and had 2014 baseline risk adjustment data.

© 2024 Roberts et al. *JAMA Health Forum.*

Study Author, Study Title, and Year	Scope/ representative ness of the ICP	Primary Data Sources	Characteristics of Dual Eligibles Studied
		Medicare risk adjustment data.	
**Kim et al, Comparing Care for Dual-Eligibles Across Coverage Models: Empirical Evidence from Oregon, 2019**	Single state	Oregon all-payer claims database with additional state Medicaid data from 2011-2014	Full-benefit duals 18 and over enrolled for at least 3-months, excluding PACE enrollees, and excluding Medicaid expansion enrollees (enrolled in 2014). Member-quarters for duals not enrolled for all three months of the quarter were excluded.
**Anderson et al, Effects of Integrating Care for Medicare-Medicaid Dually Eligible Seniors in Minnesota, 2020**	Single state	MN administrative data that included Medicare and Medicaid FFS claims, managed care encounters, and enrollment data. Years 2010-2012.	Full-benefit duals aged 65 and older for whom MSC+ was mandatory and were eligible for either MHSO or MSC+. People with IDD were excluded (b/c of eligibility through a Developmental Disabilities Waiver).
**Jung et al, Integrated Medicare and Medicaid Managed Care and Rehospitalization of Dual Eligibles, 2015**	Single state	Medicare FFS claims and commercial (MA) health plan data for 2007-2009	Mass. Duals ages 65+ without ESRD with at least 1 hospitalization and 24-months of continuous enrollment in the integrated or non-integrated plan throughout and residing in 7 counties where integrated plan was offered. Outliers with hospital length of stay above the 99th percentile (32+ days) excluded.
**Jen Associates, Massachusetts Senior Care Option 2005-2010 Impact on Enrollees: Nursing Home Entry Utilization August 14, 2013**	Single state	Medicare and Medicaid administrative data for 2004-2010	Community-dwelling Medicare-Medicaid duals in Massachusetts with at least 3 months of non-managed care baseline data prior to assignment of integrated treatment or non-integrated FFS control group. Those with pre-index

© 2024 Roberts et al. *JAMA Health Forum.*

Study Author, Study Title, and Year	Scope/ representativeness of the ICP	Primary Data Sources	Characteristics of Dual Eligibles Studied
			nursing home residence were excluded as well.

© 2024 Roberts et al. *JAMA Health Forum.*

General Study Information Part 3

Study Author, Study Title, and Year	Study Design Category	Notes on Study Design	Comparator population	Bias assessment
**Chapin et al, Program of All-inclusive Care for the Elderly (PACE) Medicaid Cost-Benefit Study, 2013**	Observational, longitudinal	Observational cohort study using a propensity score-matched comparison group	Propensity score matched sample based on demographic information (age, gender, race, and county) and LOC scores (which included cognition, ADLs, IADLs and risks). PACE was only available in 8 counties so matching was restricted to the same counties.	Risk of bias from unmeasured confounding -- study did not match on diagnosis since they were not available, and they did not employ any quasi-experimental study design.
**Segelman et al., Transitioning from Community-Based to Institutional Long-term Care: Comparing 1915(c) Waiver and PACE Enrollee 2017**	Observational, longitudinal	Observational cohort study that examines rates of admissions to LTC hospital among PACE enrollees vs. HCBS waiver cohort (comparison cohort). They used a risk proportional hazard model (death being competing risk) with state dummies and accounted for county-level clustering, individual and contextual covariates.	1915(c) aged or aged and disabled waiver group	Risk of bias from unmeasured confounding from those who enter PACE vs. those in the waiver program. There was no health status available/assessed at time of entry to program (PACE could have been healthier).
**Ghosh et al., Effects of PACE on Costs, Nursing Home Admissions, and Mortality 2006 - 2011, 2014**	Observational, longitudinal	Observational cohort study with a comparison group. Study was limited to eight states that had at least 250 new PACE enrollees and at least 3,000 new	Two propensity-matched (nearest neighbor matching) within-state control groups of Medicare beneficiaries composed of: (1) either	Used two propensity-matched control groups per state (one with new NH residents and one without NH residents) and tested for a) overlap of

© 2024 Roberts et al. *JAMA Health Forum.*

Study Author, Study Title, and Year	Study Design Category	Notes on Study Design	Comparator population	Bias assessment
		enrollees in home and community-based services (HCBS) 1915(c) waiver programs for aged and disabled individuals during 2006-2008. Outcomes were examined over successive 6-month intervals from sample entry. While Medicare expenditures were examined for a maximum of 66 months (through 2011), Medicaid expenditures were only examined for a maximum of 42 months (through 2009).	new HCBS waiver or nursing home entrants, or (2) new HCBS waiver recipients alone. Matching was based on pre-enrollment demographics, chronic conditions, utilization, and spending.	probability distributions for propensity score and b) baseline equivalence on matched characteristics. In general, groups appeared well-matched. However, there is a risk of bias from unmeasured baseline differences between cohorts that may be correlated with longer-run outcome trends.
**Wieland et al, Does Medicaid Pay More to a Program of All-Inclusive Care for the Elderly (PACE) Than for Fee-for-Service Long-term Care?, 2013**	Observational, longitudinal	A high-dimensional attrition-based regression model was used to model phenotypical differences in PACE, waiver, and NH groups and then used to predict 1-year expenditures. The PACE group was compared to the predicted fee-for-service expenditures as an alternative to long-term care compared to a blend	Comprised of duals ages 55+ in the same two SC (South Carolina) counties as PACE-enrollees and entering LTC from the community between 1994 and 2005 but enrolling in either the Medicaid community waiver program or nursing home care.	These findings are very specific to a single state and older timeframe and may not generalize to multiple states of today. In addition, the statistical models used seem more appropriate for epidemiological models related to disease and functional health phenotypes than for health care cost analyses.

Study Author, Study Title, and Year	Study Design Category	Notes on Study Design	Comparator population	Bias assessment
		of the other two groups. While the study included 11 years of data, the cost outcome only looked at 1-year expenditures for the year after admission.		
**Feng et al., Comparing Outcomes for Dual Eligible Beneficiaries in Integrated Care: Final Report, 2021**	Observational, cross-sectional	Observational, cross-sectional analysis. Multivariable regression-adjusted outcomes with state fixed effects to compare the "within-state" differences between integrated vs non-integrated plans. Given known quality issues with Medicare 2015 data, these findings should be interpreted with caution.	MA full-benefit duals enrolled in a non-D-SNP (i.e., non-integrated plan), excluding MMP and FAI-enrolled beneficiaries.	Study compared and adjusted for differences in observed patient-level demographic and clinical confounders. Unobserved confounding on patient social, functional, and cognitive risk factors is a major concern.
**Griffin et al., MyCare Ohio: Third Evaluation Report, October 2023**	Quasi-experimental	Difference-in-differences analysis comparing those eligible for the MyCare Ohio demonstration (beneficiaries living in 29 counties) to a propensity score-weighted comparison sample of beneficiaries who were ineligible for the demonstration (living in 39 counties in 14 MSAs	Propensity score-weighted comparison group: dual eligible beneficiaries living in 39 counties in 14 MSAs across six states plus 46 non-metropolitan counties in Ohio that did not participate in the demonstration. Propensity score weights balanced on individual-level characteristics such as age,	Researchers tested whether trends in Medicare Part A and B spending evolved similarly in the treatment vs. comparison groups at baseline (parallel trends test). Figures showed no violation of parallel pre-trends assumption for spending. Tests for other outcomes not reported.

© 2024 Roberts et al. *JAMA Health Forum.*

Study Author, Study Title, and Year	Study Design Category	Notes on Study Design	Comparator population	Bias assessment
		across six states plus 46 non-metropolitan counties in Ohio that did not participate in the demonstration). The difference-in-differences analysis uses an intention-to-treat (ITT) approach.	gender, disability, and HCC risk score, and on area-level characteristics such as household education level and proximity to hospitals and nursing homes.	Risk of bias is from unmeasured, time varying factors that differentially affected the treatment vs. comparison groups. This is acknowledged.
**Snow et al. Financial Alignment Initiative New York Fully Integrated Duals Advantage for Individuals with Intellectual and Developmental Disabilities: Preliminary Third Evaluation Report. 2023.**	Quasi-experimental	Difference-in-differences analysis comparing those eligible for the New York FIDA-IDD demonstration (beneficiaries living in 9 downstate counties in New York City and surrounding metropolitan area) to a propensity score-weighted comparison sample of beneficiaries with intellectual and developmental disabilities living in 12 non-rural metropolitan statistical areas within New York State (comparison areas did not participate in the demonstration). The comparison group was limited to beneficiaries in Intermediate Care Facilities or in a New York	Propensity score-weighted comparison group: dual eligible beneficiaries with intellectual and developmental disabilities living in 12 non-rural MSAs in New York that did not participate in the demonstration. The comparison group was limited to beneficiaries in Intermediate Care Facilities or in a New York State Medicaid waiver program administered by the Office for People with Developmental Disabilities. Propensity score weights balanced on individual-level characteristics such as age, gender, disability, and HCC risk score, and on	Researchers tested whether trends in Medicare Part A and B and Medicaid spending evolved similarly in the treatment vs. comparison groups at baseline (parallel trends test). Figures showed no violation of parallel pre-trends assumption for spending. However, a large, temporary divergence in post-demonstration trends was observed in 2020, which likely reflects impacts of the COVID-19 public health emergency rather than a program effect.<br><br>Risk of bias is from unmeasured, time varying

Study Author, Study Title, and Year	Study Design Category	Notes on Study Design	Comparator population	Bias assessment
		State Medicaid waiver program administered by the Office for People with Developmental Disabilities. The difference-in-differences analysis uses an intention-to-treat (ITT) approach.	area-level characteristics such as household education level and proximity to hospitals and nursing homes.	factors that differentially affected the treatment vs. comparison groups. This is acknowledged.<br><br>An additional limitation of the analysis is the low participation rate in the FIDA-IDD among dual eligible beneficiaries. This may limit the ability to isolate model effects on spending trends.
**Holladay et al., Illinois Medicare-Medicaid Alignment Initiative: Third Evaluation Report, November 2022**	Quasi-experimental	Difference-in-differences analysis comparing those eligible for the Illinois MMP demonstration (living in 21 Illinois counties that participated in the demonstration) to a propensity score-weighted comparison sample of those ineligible for the demonstration (drawn from 28 metropolitan statistical areas in 10 states). This report summarized quantitative results of the program before its statewide expansion in 2021. The	Propensity score-weighted comparison group: dual eligible beneficiaries living in 28 metropolitan statistical areas in 10 states.	Researchers tested whether trends in Medicare Part A and B spending evolved similarly in the treatment vs. comparison groups at baseline (parallel trends test). Figures showed no violation of parallel pre-trends assumption for spending. Tests for other outcomes not reported.<br><br>Risk of bias is from unmeasured, time varying factors that differentially affected the treatment vs.

© 2024 Roberts et al. *JAMA Health Forum.*

Study Author, Study Title, and Year	Study Design Category	Notes on Study Design	Comparator population	Bias assessment
		difference-in-differences analysis uses an intention-to-treat (ITT) approach.		comparison groups. This is acknowledged.
**Chepaitis et al., Virginia Commonwealth Coordinated Care Evaluation Report, Spring 2021**	Quasi-experimental	Difference-in-differences analysis comparing those eligible for the Virginia Coordinated Care MMP demonstration (living in 114 Virginia counties that participated in the demonstration [both urban and rural counties]) to a propensity score-weighted comparison sample of those ineligible for the demonstration (drawn from 186 counties in 5 states). Difference-in-differences design was only used to examine changes in Medicare spending associated with Virginia's MMP. The difference-in-differences analysis uses an intention-to-treat (ITT) approach.	Propensity score-weighted comparison group: dual eligible beneficiaries living in 186 counties in 5 states.	Researchers tested whether trends in Medicare Part A and B spending evolved similarly in the treatment vs. comparison groups at baseline (parallel trends test). Figures showed no violation of parallel pre-trends assumption for spending. Tests for other outcomes not reported.<br><br>Risk of bias is from unmeasured, time varying factors that differentially affected the treatment vs. comparison groups. This is acknowledged.
**Howard et al., South Carolina Healthy**	Quasi-experimental	Difference-in-differences analysis comparing those	Propensity score-weighted comparison group: dual	Researchers tested whether trends in

© 2024 Roberts et al. *JAMA Health Forum.*

Study Author, Study Title, and Year	Study Design Category	Notes on Study Design	Comparator population	Bias assessment
**Connections Prime Third Evaluation Report, December 2023**		eligible for the South Carolina Healthy Connections Prime MMP demonstration (living in 43 South Carolina counties that participated in the demonstration [both urban and rural counties]) to a propensity score-weighted comparison sample of those ineligible for the demonstration (drawn from 51 counties in 5 states). The difference-in-differences analysis uses an intention-to-treat (ITT) approach.	eligible beneficiaries living in 51 counties in 5 states.	Medicare Part A and B spending evolved similarly in the treatment vs. comparison groups at baseline (parallel trends test). Figures showed no violation of parallel pre-trends assumption for spending. Tests for other outcomes not reported.<br><br>Risk of bias is from unmeasured, time varying factors that differentially affected the treatment vs. comparison groups. This is acknowledged.
**Gattine et al., Financial Alignment Initiative Massachusetts One Care: Preliminary Fifth Evaluation Report**	Quasi-experimental	Difference-in-differences analysis comparing those eligible for the Massachusetts OneCare demonstration (living in counties that participated in the demonstration to a propensity score-weighted comparison sample of those ineligible for the demonstration (drawn from 115 nonparticipating counties in 24 MSAs). The difference-in-differences	Propensity score-weighted comparison group: dual eligible beneficiaries living in 115 counties in 24 MSAs not part of the demonstration.	Researchers tested whether trends in Medicare Part A and B spending and utilization evolved similarly in the treatment vs. comparison groups at baseline (parallel trends test). They find some violations of the parallel trends assumption for Medicare spending, inpatient use, SNF use, and hospital admissions for ambulatory care

© 2024 Roberts et al. *JAMA Health Forum.*

Study Author, Study Title, and Year	Study Design Category	Notes on Study Design	Comparator population	Bias assessment
		analysis uses an intention-to-treat (ITT) approach.		conditions. To address this, the researchers ran alternative models that adjusted for differential pre-trends (the assumption is that such differences in trends would have persisted absent the demonstration). They did not find meaningfully different results.
**Holladay et al., Financial Alignment Initiative Michigan MI Health Link Second Evaluation Report, March 2022**	Quasi-experimental	Difference-in-differences analysis comparing those eligible for the Michigan HealthLink demonstration (living in counties that participated in the demonstration (upper peninsula, SW Michigan, and Detroit metro area) to a propensity score-weighted comparison sample of those ineligible for the demonstration (drawn from 40 nonparticipating counties in Michigan and 18 counties from 4 other states). The difference-in-differences analysis uses	Propensity score-weighted comparison group: dual eligible beneficiaries living in 58 nonparticipating counties (40 in Michigan and 18 counties from 4 other states).	Researchers tested whether trends in Medicare Part A and B spending evolved similarly in the treatment vs. comparison groups at baseline (parallel trends test). Figures showed no violation of parallel pre-trends assumption for spending. Tests for other outcomes not reported.<br><br>Risk of bias is from unmeasured, time varying factors that differentially affected the treatment vs. comparison groups. This is acknowledged.

© 2024 Roberts et al. *JAMA Health Forum.*

Study Author, Study Title, and Year	Study Design Category	Notes on Study Design	Comparator population	Bias assessment
		an intention-to-treat (ITT) approach.		
**Gattine et al. Rhode Island Integrated Care Initiative: Third Evaluation Report, December 2023**	Quasi-experimental	Difference-in-differences analysis comparing those eligible for the Rhode Island Integrated Care Initiative (living in any of the state's 5 counties) to a propensity score-weighted comparison sample of those ineligible for the demonstration (drawn from 17 counties in 2 states). The difference-in-differences analysis uses an intention-to-treat (ITT) approach.	Propensity score-weighted comparison group: dual eligible beneficiaries living in 17 counties in 2 control states.	Researchers tested whether trends in Medicare Part A and B spending evolved similarly in the treatment vs. comparison groups at baseline (parallel trends test). Figures showed no violation of parallel pre-trends assumption for spending. Tests for other outcomes not reported.<br><br>Risk of bias is from unmeasured, time varying factors that differentially affected the treatment vs. comparison groups. This is acknowledged.
**Griffin et al., Texas Dual Eligible Integrated Care Demonstration: Preliminary Third Evaluation Report, December 2023**	Quasi-experimental	Difference-in-differences analysis comparing those eligible for the Texas demonstration (living in 6 counties that participated in the demonstration) to a propensity score-weighted comparison sample of those ineligible for the demonstration (drawn	Propensity score-weighted comparison group: dual eligible beneficiaries living in 7 states.	Researchers tested whether trends in Medicare spending and Medicaid evolved similarly in the treatment vs. comparison groups at baseline (parallel trends test). Their analyses found parallel Medicare spending trends but

© 2024 Roberts et al. *JAMA Health Forum.*

Study Author, Study Title, and Year	Study Design Category	Notes on Study Design	Comparator population	Bias assessment
		from 7 states). The difference-in-differences analysis uses an intention-to-treat (ITT) approach.		differential Medicaid spending trends in the baseline period. For Medicaid spending outcomes, analyses were run using an in-state comparison sample to balance on preintervention trends.<br><br>Risk of bias is from unmeasured, time varying factors that differentially affected the treatment vs. comparison groups. This is acknowledged.
**Khatutsky et al, California Cal MediConnect Preliminary Third Evaluation Report, April 2023**	Quasi-experimental	Difference-in-differences analysis comparing those eligible for the Cal MediConnect demonstration (living in 7 counties that participated in the demonstration) to a propensity score-weighted comparison sample of those ineligible for the demonstration (drawn from 11 states). The difference-in-differences analysis uses an intention-to-treat (ITT) approach.	Propensity score-weighted comparison group: dual eligible beneficiaries living in 11 states (168 urban counties in 10 states and 40 non-urban counties in Michigan).	Researchers tested whether Medicare spending trends evolved similarly in the treatment vs. comparison groups at baseline (parallel trends test). Their analyses found parallel Medicare spending trends at baseline.<br><br>Risk of bias is from unmeasured, time varying factors that differentially affected the treatment vs.

© 2024 Roberts et al. *JAMA Health Forum.*

Study Author, Study Title, and Year	Study Design Category	Notes on Study Design	Comparator population	Bias assessment
				comparison groups. This is acknowledged.<br><br>Note that the only quasi-experimental difference-in-differences analysis is on Medicare spending.
**Caswell et al., Do Integrated Care Models for Dual Medicare-Medicaid Enrollees Work? Evidence from Massachusetts' One Care Financial Alignment Demonstration (In press, 2023)**	Quasi-experimental	This is an intention-to-treat regression discontinuity design that compares individuals just below the age eligibility ceiling (<65 years) for OneCare vs. those just above this eligibility ceiling at the start of the demonstration.	Dual eligible individuals above the age eligibility ceiling for OneCare (an age bandwidth of 84 months, or 7 years, above and below age 65 is used to delimit the population included in the regression discontinuity model)	Assumption of regression discontinuity design is that confounders trend smoothly across the age cutoff. Authors do report covariate balance for observed variables. There are some imbalances (e.g., on age) though this is partly mechanical because of the age cutoff employed in the RD design.
**Chen et al, Early evidence from South Carolina's Medicare-Medicaid dual-eligible financial alignment initiative: an observational study to understand who enrolled, and whether the program improved health, 2018**	Observational, longitudinal	Longitudinal cohort analysis, which compared dual eligibles in South Carolina who ever vs. never joined Healthy Connections Prime (South Carolina's MMP). 'Ever joined' is defined as enrolled in the program for at least 6 months in the study window. Event-study analysis compares	Dual eligible individuals who did not join Healthy Connections Prime (South Carolina's MMP)	Design compared dual eligibles in South Carolina who ever vs. never joined Healthy Connections Prime (South Carolina's MMP). Design relies on the strong assumption that enrollment in Healthy Connections Prime is unrelated to changes in health care use or health status that would drive

© 2024 Roberts et al. *JAMA Health Forum.*

Study Author, Study Title, and Year	Study Design Category	Notes on Study Design	Comparator population	Bias assessment
		changes in the ever-joined group to the never-joined group.		utilization trends. Limitations of this assumption were not discussed. Event-study graphs reveal slight pre-trend differences in SNF use though not hospitalizations or ED visits.
**Graham et al., Beneficiaries Respond To California's Program to Integrate Medicare, Medicaid, And Long-Term Services, 2018**	Observational, cross-sectional	Cross-sectional survey sample of CMC enrollees that remained in CMC in 7 demonstration counties and a random phone survey sample of duals from 9 counties that matched the demonstration counties.	Random survey sample of duals in 9 non-demonstration counties that had long-term Medi-Cal managed care infrastructures similar to the demonstration counties (n=474). Of note, they also sampled CMC duals who opted out of the program (but results were not shown in an effort to "provide the cleanest comparison").	Bias related to responders vs. non-responders (though 65% response rate); limited sample (sample size of 744 in a program of ~120K duals). Bias related to unmeasured confounding from duals in demonstration counties vs. those in non-demonstration counties. Exclusion of CMC opt-outs also introduced bias (since they were sex, race/ethnicity, and disability status differences relative to those who remained). Sample is also not representative of general CMC population because of how survey was

© 2024 Roberts et al. *JAMA Health Forum.*

Study Author, Study Title, and Year	Study Design Category	Notes on Study Design	Comparator population	Bias assessment
				conducted (underrepresents those living in NFs and those who did not speak English/Spanish or sign language). Study was limited to within group comparisons of beneficiaries' changing experiences in the post-enrollment period (and did not use a diff-in-diff approach).
**Meyers et al., Medicare and Medicaid Dual-Eligible Special Needs Plan Enrollment and Beneficiary-Reported Experiences with Care, 2023**	Observational, cross-sectional	Observational cross-sectional study. Analysis used multivariable linear regressions (adjusting for respondent characteristics, OREC, self-reported physical/mental health status, state-fixed effects; and use of a proxy as respondent; with plan random effects) to compare patient satisfaction scores across 3 groups: FIDEs, Coordinating only D-SNPs, and regular MA plans.	Two groups: Full benefit duals in Coordination-only D-SNPs and those in conventional non-D-SNP MA plans	Bias related to unmeasured confounding; limited generalizability to non-responders (45% response rate). Observational, cross-sectional analysis with no quasi-experimental study design.

© 2024 Roberts et al. *JAMA Health Forum.*

Study Author, Study Title, and Year	Study Design Category	Notes on Study Design	Comparator population	Bias assessment
**Roberts et al., Changes in care associated with integrating Medicare and Medicaid for dual eligible individuals, In Press 2023**	Observational, longitudinal	Longitudinal cohort study with a propensity score weighted comparison group (analyzed using difference-in-differences analysis). The design compared changes in care between two cohorts of dual-eligible beneficiaries: 1) a cohort whose coverage became integrated in Medicare and Medicaid managed care plans operated by the same insurer in 2018, and 2) a comparison cohort of dual-eligible beneficiaries who remained enrolled in fee-for-service Medicare before and after the introduction of Medicaid managed care (i.e., nonintegrated coverage). Analysis is done at the person-month level and compares changes across the cohorts before vs. after the introduction of integrated coverage in the treatment group to contemporaneous changes	Propensity score-weighted 'comparison cohort' of dual-eligible beneficiaries who remained enrolled in fee-for-service Medicare before and after the introduction of Medicaid managed care. The comparison cohort consisted of enrollees in UPMC's Medicaid managed care plan (introduced in 2018) but who continued to receive nonintegrated coverage in fee-for-service Medicare. Comparison cohort was drawn from the same counties in Southwestern Pennsylvania where members of the integration cohort lived. Propensity score weighting accounted for baseline age, sex, race and ethnicity, disability, comorbidities, and residential context (e.g., Area Deprivation Index and rurality)	To test for unmeasured factors that could lead to differential outcome trends in the absence of integration, the researchers compared 1) pre-integration outcome trends, and 2) post-integration differences in survival rates, between the integration and comparison cohorts. Differential survival rates early in the post-integration period might reflect the influence of unmeasured risk factors preceding integration, which might not be picked up in a comparison of pre-integration trends because the sample was limited to survivors over the pre-integration period.<br><br>The authors identified differential mortality rates early in the post-period and found that estimates for some outcomes (e.g., long-term nursing home

© 2024 Roberts et al. *JAMA Health Forum.*

Study Author, Study Title, and Year	Study Design Category	Notes on Study Design	Comparator population	Bias assessment
		in the comparison group. Both cohorts were followed, while alive, through 2020.		stays) were sensitive to these mortality differences. These findings suggest that some study estimates were sensitive to bias from unmeasured baseline risk factors.
**Keohane et al., Aligning Medicaid and Medicare Advantage Managed Care Plans for Dual-Eligible Beneficiaries, 2021**	Quasi-experimental	The model was a fixed-effects model to assess the association between increased county-level participation in aligned benefits and healthcare utilization of all duals. Data were analyzed as serial cross-sections. The unit of observation was person-month for observations with main exposure being % duals enrolled in aligned plan within a county. Variable was lagged by 6months (so growth of aligned plan in prior 6mo to ensure variable preceded recent changes in aligned plan	Full-benefit duals in non-aligned D-SNPs, TM, or non-DNP MA plans	Bias from unmeasured confounding (specifically, unmeasured time-varying factors correlated with changes in county-level penetration of aligned enrollment); at risk for ecological fallacy given that main models were assessing % growth of aligned D-SNP within county to overall county utilization of services. Exposure period preceded outcome observations by 6mo to help ensure that directionality of observations was growth of MA affecting changes in utilization.

© 2024 Roberts et al. *JAMA Health Forum.*

Study Author, Study Title, and Year	Study Design Category	Notes on Study Design	Comparator population	Bias assessment
		participation). County fixed effects included to control for time-variant and local county characteristics. Monthly fixed effects were also included. Generalized linear models with a Poisson and binomial distribution were used for counting outcomes and binary outcomes, respectively. We classify this as quasi-experimental because the exposure variable (county-level participation in aligned plans) is supposed to reflect policy-driven factors that increase the availability of such plans to dual-eligible beneficiaries. A version of difference in difference analysis with two-way fixed effects was used.		
**Feng et al., Comparing Outcomes for Dual Eligible Beneficiaries in Integrated Care: Final Report, 2021**	Observational, cross-sectional	Multivariable regression-adjusted outcomes with state fixed effects to compare the "within-state" differences between	MA full-benefit duals enrolled in a non-D-SNP (i.e., non-integrated plan), excluding MMP and FAI-enrolled beneficiaries.	Study compared and adjusted for differences in observed patient-level demographic and clinical confounders. Unobserved

Study Author, Study Title, and Year	Study Design Category	Notes on Study Design	Comparator population	Bias assessment
		integrated vs non-integrated plans. Given known quality issues with Medicare 2015 data, these findings should be interpreted with caution.		confounding on patient social, functional, and cognitive risk factors is a major concern.
**Kim et al, Comparing Care for Dual-Eligibles Across Coverage Models: Empirical Evidence from Oregon, 2019**	Observational, longitudinal	Observational, longitudinal study (uses serial cross-sectional data)	All other Oregon full-benefit duals in managed care or fee-for-service as the non-aligned comparator	Differences in patient-level observed demographic and clinical confounders were compared and adjusted for. Unobserved confounding outside of administrative data is a major concern.
**Anderson et al, Effects of Integrating Care for Medicare-Medicaid Dually Eligible Seniors in Minnesota, 2020**	Observational, cross-sectional	Observational, cross-sectional study. Used propensity score weighting methods to adjusted for observed differences between MSHO and MSC+ enrollees (demographics, health and disability status, chronic conditions, and county-level SES variables like % married, education, % who do not live in community)	Control were duals enrolled in MN Senior Care Plus (MSC+), a Medicaid-only managed care plan with Medicare FFS. Propensity score weighting was used. N=21,935.	Bias due to unmeasured confounding and lack of quasi-experimental study design. To address this, the authors conducted a sensitivity analysis that simulated bias from unmeasured variables (using a method proposed by Oster). The study reports bias-adjusted estimates and evaluates whether findings are robust across the main and bias-adjusted estimates.
**Jung et al, Integrated Medicare and Medicaid**	Observational, longitudinal	Observational, longitudinal study (uses	FFS duals ages 65+ without ESRD residing in	In addition to testing and adjusting for observed

© 2024 Roberts et al. *JAMA Health Forum.*

Study Author, Study Title, and Year	Study Design Category	Notes on Study Design	Comparator population	Bias assessment
**Managed Care and Rehospitalization of Dual Eligibles, 2015**		serial cross-sectional data). The comparison group consisted of dual-eligible beneficiaries residing in the same counties as Massachusetts Senior Care Options (SCO) enrollees but who had nonintegrated, fee-for-service Medicare enrollment.	7 Mass counties where integrated managed care plan was offered with consistent coverage throughout the study period.	demographic and clinical differences, the authors did a lot of sensitivity analyses, including looking at COPD and HF-specific rehospitalization as well as a difference in difference analysis. Of note, there are no county fixed effects in the OR models.
**Jen Associates, Massachusetts Senior Care Option 2005-2010 Impact on Enrollees: Nursing Home Entry Utilization August 14, 2013**	Observational, longitudinal	Longitudinal cohort study using a matched comparison cohort. Cox proportional hazards models were used to compare the mean time to long-stay NH entry, short-stay entry, and death in the treatment (integrated plan) vs control (non-integrated FFS) groups.	Community-dwelling FFS duals that were directly matched to the integrated plan duals on key demographic and insurance and clinical characteristics and then propensity-matched on other characteristics -- there were roughly 3 controls for every case. Those with pre-index nursing home residence were excluded.	Direct matching helped ensure baseline equivalence on many characteristics and the authors conducted additional analyses to show baseline equivalence on matched characteristics. However, unmeasured differences between groups at baseline could be correlated with long-run utilization and survival trends, which could have biased the findings.

© 2024 Roberts et al. *JAMA Health Forum.*

Medicare Spending

Study Author, Study Title, and Year	Findings regarding Medicare spending	Free text notes (succinct summary of findings)
**Ghosh et al., Effects of PACE on Costs, Nursing Home Admissions, and Mortality 2006 - 2011, 2014**	No change	Compared to the matched comparison group of both new HCBS waiver enrollees and NH entrants, capitated Medicare spending among PACE enrollees was similar to predicted FFS across successive 6-month intervals, with significant differences observed in only a few periods (including the first six months after enrollment, during which PACE enrollees had relatively lower Medicare expenditures).
**Griffin et al., MyCare Ohio: Third Evaluation Report, October 2023**	Increased	MyCare Ohio demonstration was associated with a differential increase in Medicare Parts A and B spending of $77.79, per member per month (PMPM), in the treatment vs. comparison groups over approximately 6.5 demonstration years.
**Snow et al. Financial Alignment Initiative New York Fully Integrated Duals Advantage for Individuals with Intellectual and Developmental Disabilities: Preliminary Third Evaluation Report. 2023.**	No change	Over the demonstration period (approximately 4.75 years), Medicare Parts A and B spending did not change significantly between the treatment vs. comparison groups. (The difference-in-differences estimates could not rule out relative spending increases as large as +$48 per member per month or relative spending reductions as large as -$25 per member per month.)
**Holladay et al., Illinois Medicare-Medicaid Alignment Initiative: Third Evaluation Report, November 2022**	Increased	Illinois MMP demonstration was associated with a differential increase in Medicare Parts A and B spending of $73.67, per member per month (PMPM), in the treatment vs. comparison groups over approximately 5 demonstration years.
**Chepaitis et al., Virginia Commonwealth Coordinated Care Evaluation Report, Spring 2021**	Increased	Virginia Commonwealth Coordinated Care MMP demonstration was associated with a differential increase in Medicare spending of $95.67, per member per month *PMPM), in the treatment vs. comparison group over approximately 4 demonstration years.
**Howard et al., South Carolina Healthy Connections Prime Third Evaluation Report, December 2023**	Increased	South Carolina Healthy Connections Prime was associated with a differential increase in Medicare Parts A and B spending of $46.14,

© 2024 Roberts et al. *JAMA Health Forum.*

		per member per month (PMPM) in the treatment vs. comparison group over approximately 6 demonstration years.
**Gattine et al., Financial Alignment Initiative Massachusetts One Care: Preliminary Fifth Evaluation Report**	Increased	Over approximately 6 demonstration years, the cumulative demonstration effect was an increase in Medicare Parts A and B expenditures for all eligible beneficiaries ($36.98 per member per month) in the treatment vs. comparison groups. The demonstration did not significantly affect Medicare expenditures in years 1-2 but was associated with increased spending in years 3-6.
**Holladay et al., Financial Alignment Initiative Michigan MI Health Link Second Evaluation Report, March 2022**	Increased	Over approximately 4 demonstration years, Medicare spending increased by $118.05 per member per month in the treatment vs. comparison groups.
**Gattine et al. Rhode Island Integrated Care Initiative: Third Evaluation Report, December 2023**	Increased	Over 4.5 demonstration years, Medicare spending increased differentially by $83.99 per member per month in the treatment vs. comparison groups.
**Griffin et al., Texas Dual Eligible Integrated Care Demonstration: Preliminary Third Evaluation Report, December 2023**	No change	Over approximately 5 demonstration years, there was no significant change in Medicare spending between the treatment vs. comparison groups.
**Khatutsky et al, California Cal MediConnect Preliminary Third Evaluation Report, April 2023**	Increased	Over approximately 6 years of the demonstration (April 2014 - December 2019), Medicare spending increased by an average of $62.82 per member per month in the treatment vs. comparison groups.

© 2024 Roberts et al. *JAMA Health Forum.*

Medicaid Spending

Study Author, Study Title, and Year	Findings regarding Medicaid spending	Free text notes (succinct summary of findings)
**Chapin et al, Program of All-inclusive Care for the Elderly (PACE) Medicaid Cost-Benefit Study, 2013**	Decreased	Key Finding 1: PACE had lower Medicaid expenditures than a matched control group of customers in nursing facilities (4-yr weighted average monthly savings of over $1,000 (38%) per customer). However, relative to the 2nd control group (HCBS/FE), PACE cost on average $320 more per person per month over a 4-hr weighted aggregated period. I still checked off decreased given the finding relative to those in NFs (since the HCBS/FE waiver group is arguably a different type of control). Key Finding 2: PACE Medicaid expenditures were significantly lower than both controls HCBS/FE and NF during 3-mo period before death (by 43% and 33%, respectively, saving $3,907 and $2,594).
**Ghosh et al., Effects of PACE on Costs, Nursing Home Admissions, and Mortality 2006 - 2011, 2014**	Increased	Compared to the matched comparison group of both new HCBS waiver enrollees and NH entrants, capitated Medicaid spending among PACE enrollees exceeded predicted FFS expenditures overall, with some heterogeneity over time and across states. Over the first five intervals post-enrollments, Medicaid expenditures among PACE enrollees exceeded predicted FFS expenditures. After this period, differences were still positive, but were 30-40% smaller than in the 25–30-month post-enrollment interval and were not statistically significant. Notable state-specific findings included significantly lower Medicaid expenditures among PACE enrollees in NY, higher Medicaid spending in California (with the gap increasing over the first four intervals), and a smaller positive spending gap in Massachusetts that decreased over time.
**Wieland et al, Does Medicaid Pay More to a Program of All-Inclusive Care for the Elderly (PACE) Than for**	Decreased	Full-year Medicaid capitation for PACE was $27,648 in FY05 — 28% below the lower limit of case-mix adjusted fee-for-service payments in LTC. In FY05 dollars, this

**Fee-for-Service Long-term Care?, 2013**		amounted to more than $8.5 million in savings in the first year for beneficiaries admitted to PACE over the 11-year study period.
**Snow et al. Financial Alignment Initiative New York Fully Integrated Duals Advantage for Individuals with Intellectual and Developmental Disabilities: Preliminary Third Evaluation Report. 2023.**	No change	Over the demonstration period (approximately 4.75 years), Medicaid spending did not change significantly between the treatment vs. comparison groups. (However, the difference-in-differences estimates were imprecise and could not rule out relative spending increases as large as +$725 per member per month or relative spending reductions as large as -$885 per member per month.)
**Gattine et al., Financial Alignment Initiative Massachusetts One Care: Preliminary Fifth Evaluation Report**	Increased	Over approximately 6 demonstration years, the cumulative demonstration effect was an increase in Medicaid expenditures for all eligible beneficiaries ($129.02 per member per month). The demonstration was associated with increased Medicaid expenditures in years 2-4 and 6; in years 1 and 5, there was no statistically significant effect.
**Griffin et al., Texas Dual Eligible Integrated Care Demonstration: Preliminary Third Evaluation Report, December 2023**	No change	Over approximately 6 demonstration years, the demonstration was not associated with a significant reduction in Medicaid spending per member per month. This analysis was performed using a within-state comparison group that exhibited better balance on baseline trends than the out-of-state comparison group used in other analyses.
**Khatutsky et al, California Cal MediConnect Preliminary Third Evaluation Report, April 2023**	Increased	Over approximately 6 years of the demonstration (April 2014 - December 2019), Medicaid spending increased by an average of $325.46 per member per month in the treatment vs. comparison groups.<br><br>Note: The researchers found some potentially aberrant changes in Medicaid spending in the post-period (approximately 35-50 months post-implementation of Cal MediConnect) and dropped Medicaid spending data from those months from their difference-in-differences analysis.

© 2024 Roberts et al. *JAMA Health Forum.*

© 2024 Roberts et al. *JAMA Health Forum.*

Long-term Nursing Home Stays

Study Author, Study Title, and Year	Findings regarding long-term nursing home stays	Free text notes (succinct summary of findings)
**Chapin et al, Program of All-inclusive Care for the Elderly (PACE) Medicaid Cost-Benefit Study, 2013**	No change	Percent admitted to LT NF stays in PACE was 15.44% vs. 15.07% in HCBS/FE (note: NF people not in this comparison). Long term stays were defined as stays of 90 days or more.
**Segelman et al., Transitioning from Community-Based to Institutional Long-term Care: Comparing 1915(c) Waiver and PACE Enrollee 2017**	Decreased	PACE cohort had lower unadjusted rates of long-term NH admission than those in HCBS waiver (12.76% vs. 16.90%, $p<0.001$). PACE had 31% lower adjusted risk of long-term NH admission. At admission, PACE enrollees were overall more cognitively impaired (0.34 points, $p<0.001$) and had 55% higher odds (CPS>=4) of severe cognitive impairment and 45% higher odds of having overall high impairment ($p=0.003$). This potentially suggests that PACE may be better at reducing LT NH stays and keep people with more impairment in the community longer.
**Ghosh et al., Effects of PACE on Costs, Nursing Home Admissions, and Mortality 2006 - 2011, 2014**	Decreased	Comparing PACE enrollees to the matched comparison group that only included HCBS waiver enrollees and not NH entrants, PACE enrollees were more likely to use a NH in each of the seven intervals examined, with statistically significant differences in the first four intervals. However, the proportion of days in the NH and the likelihood of being in a NH for at least 30 days was similar across the treatment and comparison groups. Additionally, relative to the comparison group, PACE enrollees were approximately 2-4 percentage points less likely to be in a NH for at least 90 days in any particular interval, but the cumulative rates of being in a NH for at least 90 days was similar across the treatment and comparison groups across intervals (difference in findings reflects the extent to which nursing home stays overlapped). When comparing PACE enrollees to the comparison group comprised of both waiver enrollees and NH entrants, PACE enrollees (as

© 2024 Roberts et al. *JAMA Health Forum.*

		expected) had significantly lower utilization of NH services across all NH utilization outcomes.
**Feng et al., Comparing Outcomes for Dual Eligible Beneficiaries in Integrated Care: Final Report, 2021**	Decreased	Compared to enrollees in non-integrated MA plans, PACE enrollees were much less likely to have any institutional use (Medicaid-covered stays in a nursing facility, intermediate care facility, or inpatient psychiatric hospital) in 2015 (OR = 0.062; $p < 0.001$).
**Griffin et al., MyCare Ohio: Third Evaluation Report, October 2023**	Decreased	Over approximately 6.5 years, the annual probability of any long-term nursing home use declined by 4.2 percentage points in the treatment vs. comparison groups.
**Holladay et al., Illinois Medicare-Medicaid Alignment Initiative: Third Evaluation Report, November 2022**	Increased	Over approximately 5 years, the annual probability of any long-term nursing home used increased by 3.78 percentage points in the treatment vs. comparison groups
**Howard et al., South Carolina Healthy Connections Prime Third Evaluation Report, December 2023**	Decreased	Over approximately 6 years, the annual probability of any long-term nursing home used decreased by 1.46 percentage points in the treatment vs. comparison groups.
**Gattine et al., Financial Alignment Initiative Massachusetts One Care: Preliminary Fifth Evaluation Report**	Decreased	Over approximately 6 demonstration years, there was a 0.54 percentage point reduction in annual probability of any long-stay nursing facility use in the treatment vs. comparison groups
**Holladay et al., Financial Alignment Initiative Michigan MI Health Link Second Evaluation Report, March 2022**	Increased	Over approximately 4 demonstration years, the annual probability of nursing home use increased by 1.4 percentage points in the treatment vs. comparison groups.
**Gattine et al. Rhode Island Integrated Care Initiative: Third, December 2023**	No change	Over 4.5 demonstration years, the annual probability of any long-stay nursing home use did not change significantly between the treatment vs. comparison groups.
**Griffin et al., Texas Dual Eligible Integrated Care Demonstration: Preliminary Third Evaluation Report, December 2023**	Decreased	Over approximately 6 demonstration years, the annual probability of long-stay nursing home use decreased by approximately 1.82 percentage points more in the treatment group relative to the comparison group, corresponding to a relative decrease of 10.1% in

© 2024 Roberts et al. *JAMA Health Forum.*

		the probability of any long-stay nursing home use among the demonstration group during the demonstration period.
**Caswell et al., Do Integrated Care Models for Dual Medicare-Medicaid Enrollees Work? Evidence from Massachusetts' One Care Financial Alignment Demonstration (In press, 2023)**	No change	No discontinuity in long-term nursing facility use (extensive margin of any, or intensive margin of days) across the age eligibility threshold for OneCare.
**Roberts et al., Changes in care associated with integrating Medicare and Medicaid for dual eligible individuals, In Press 2023**	Inconclusive/mixed	Inclusive (overall), as estimates of changes in long-term nursing home stays were sensitive to mortality differences early in the post-integration period. Descriptive analyses provided some support for lower long-stay institutional care use among individuals initially receiving HCBS, although these analyses did not include a comparison to pre-integration trends
**Keohane et al., Aligning Medicaid and Medicare Advantage Managed Care Plans for Dual-Eligible Beneficiaries, 2021**	Decreased	They used TennCare Medicaid encounter data to determine nursing home users so assuming this is LT NH stay. For a 10%-point increase in aligned plan participation, there was a -0.3 (-0.6, 0.0) change in nursing home users per month per 100 beneficiaries. Only reported for benes 65+.
**Feng et al., Comparing Outcomes for Dual Eligible Beneficiaries in Integrated Care: Final Report, 2021**	Decreased	Compared to enrollees in non-integrated MA plans, FIDE-SNP enrollees were much less likely to have any institutional use (Medicaid-covered stays in a nursing facility, intermediate care facility, or inpatient psychiatric hospital) in 2015 (OR = 0.320; p < 0.001).
**Anderson et al, Effects of Integrating Care for Medicare-Medicaid Dually Eligible Seniors in Minnesota, 2020**	No change	Estimate of MSHO effect of any long-term NF stay was 0.004 (-0.003, 0.012, p=0.235).
**Jen Associates, Massachusetts Senior Care Option 2005-2010 Impact on Enrollees: Nursing Home Entry Utilization August 14, 2013**	Decreased	In the proportional hazards model, SCO enrollment was associated with a 16% reduction in the risk of long-term nursing facility stays (Hazards ratio of 0.84 for SCO enrollees, confidence limits 0.77 to 0.91, p value <.001). In the proportional hazards model, SCO enrollment was associated with a 23% reduction in the risk of end-

© 2024 Roberts et al. *JAMA Health Forum.*

		of-life nursing facility entrance (Hazards ratio of 0.77, confidence limits 0.67 to 0.88, p value <.001)

© 2024 Roberts et al. *JAMA Health Forum.*

HCBS

Study Author, Study Title, and Year	Findings regarding HCBS	Free text notes (succinct summary of findings)
**Caswell et al., Do Integrated Care Models for Dual Medicare-Medicaid Enrollees Work? Evidence from Massachusetts' One Care Financial Alignment Demonstration (In press, 2023)**	Increased	Home-based service days were 0.506 days greater (15%) for the treatment group—i.e., just below the age threshold.<br><br>The probability of having an assessment for HCBS was 1.44 percentage points (5%) greater for the treatment group—i.e., just below the age threshold.
**Graham et al., Beneficiaries Respond To California's Program to Integrate Medicare, Medicaid, And Long-Term Services, 2018**	No change	CMC vs. control: % using in-home supportive services not significantly different (54.7% vs. 53.9%) and hours of IHSS not significantly different (82 vs. 89.5 hours). Of note, changes in the % of respondents who reported using IHSS (in-home supportive services) was not statistically different in T1 vs. T2; though CMC duals reported statistically significant fewer hours of IHSS used per month. In the area of unmet need, Cal MediConnect recipients who needed help with personal care services were less likely to report having unmet needs (26%) compared to the comparison group (43%).
**Roberts et al., Changes in care associated with integrating Medicare and Medicaid for dual eligible individuals, In Press 2023**	Increased	From baseline through years 1-, 2-, and 3-years post-integration, use of HCBS increased differentially by 0.28, 0.49, and 0.61 days per person-month (respectively) in the integration vs. comparison cohorts. These changes represent increases of approximately 10% to 20%, relative to baseline levels in the integration cohort.
**Keohane et al., Aligning Medicaid and Medicare Advantage Managed Care Plans for Dual-Eligible Beneficiaries, 2021**	No change	For every 10%-point increase of aligned-DSNP participation, there was a 0.2 (-0.1, 0.5) change in HCBS users 65+ and 0(-0.1, 0.2) change in HCBS users <65 per month per 100 beneficiaries.
**Feng et al., Comparing Outcomes for Dual Eligible Beneficiaries in Integrated Care: Final Report, 2021**	Increased	Compared to enrollees in non-integrated MA plans, FIDE-SNP enrollees were much more likely to use HCBS in 2015 (OR = 4.223; $p < 0.001$).

© 2024 Roberts et al. *JAMA Health Forum.*

**Anderson et al, Effects of Integrating Care for Medicare-Medicaid Dually Eligible Seniors in Minnesota, 2020**	Inconsistent/mixed	In Table 3, estimate of MSHO effect of any HCBS use was significantly higher with coefficient of 0.021 (0.013, 0.03, P<0.001). However, in Table 4, after applying Oster's method to generate bias-adjusted estimates of the effects of MHSO, the estimate of MSHO on HCBS use in Table 3 would unlikely hold up if controlled for unobserved variables in analysis.

© 2024 Roberts et al. *JAMA Health Forum.*

Hospital Admissions, overall

Study Author, Study Title, and Year	Findings regarding hospital admissions, overall	Free text notes (succinct summary of findings)
**Chapin et al, Program of All-inclusive Care for the Elderly (PACE) Medicaid Cost-Benefit Study, 2013**	No change	Number of hospital admissions was not statistically significantly lower in PACE vs. HCBS vs. NF (0.60 vs. 0.73 vs. 0.78 admissions per person per year, respectively) though the number of days in hospital was significantly lower in PACE vs. HCBS and NF (2.64 vs. 4.53 vs. 4.99, respectively).
**Feng et al., Comparing Outcomes for Dual Eligible Beneficiaries in Integrated Care: Final Report, 2021**	Decreased	PACE beneficiaries were significantly less likely to be hospitalized than those in non-integrated MA plans in 2015 (OR = 0.689; $p < 0.001$).
**Griffin et al., MyCare Ohio: Third Evaluation Report, October 2023**	Decreased	Over approximately 6.5 years, the monthly probability of any inpatient admission declined by 0.57 percentage points in the treatment vs. comparison groups.
**Holladay et al., Illinois Medicare-Medicaid Alignment Initiative: Third Evaluation Report, November 2022**	Increased	Over approximately 5 years, the monthly probability of any inpatient admission increased by 0.11 percentage points in the treatment vs. comparison groups.
**Howard et al., South Carolina Healthy Connections Prime Third Evaluation Report, December 2023**	Decreased	Over approximately 6 years, the monthly probability of any inpatient admission decreased by 0.56 percentage points in the treatment vs. comparison groups.
**Gattine et al., Financial Alignment Initiative Massachusetts One Care: Preliminary Fifth Evaluation Report**	Increased	Over approximately 6 demonstration years, the monthly probability of an inpatient admission increased by 0.18 percentage points in the treatment vs. comparison groups.
**Holladay et al., Financial Alignment Initiative Michigan MI Health Link Second Evaluation Report, March 2022**	No change	Over approximately 4 years, the probability of having an inpatient admission did not change significantly between the treatment vs. comparison groups.
**Gattine et al. Rhode Island Integrated Care Initiative: Combined Third Evaluation Report, December 2023**	No change	Over 4.5 years, the annual probability of inpatient admission did not change significantly in the treatment vs. comparison groups.

© 2024 Roberts et al. *JAMA Health Forum.*

**Griffin et al., Dual Eligible Integrated Care Demonstration: Preliminary Third Evaluation Report, December 2023**	No change	Over approximately 6 years, the change in annual probability of an inpatient admission did not differ significantly in the treatment vs. comparison groups.
**Caswell et al., Do Integrated Care Models for Dual Medicare-Medicaid Enrollees Work? Evidence from Massachusetts' One Care Financial Alignment Demonstration (In press, 2023)**	No change	No statistically significant discontinuity in hospital admissions across the age eligibility threshold for OneCare.
**Chen et al, Early evidence from South Carolina's Medicare-Medicaid dual-eligible financial alignment initiative: an observational study to understand who enrolled, and whether the program improved health, 2018**	No change	No differential change in inpatient admissions after 5 quarters between program participants and eligible non-participants. Study also found no differential change in inpatient length of stay (conditional on admission)
**Graham et al., Beneficiaries Respond To California's Program to Integrate Medicare, Medicaid, And Long-Term Services, 2018**	No change	CMC vs. control: 0.63 vs. 0.37 overnight hospital stays per person in last 6mo from survey date (not significant).
**Roberts et al., Changes in care associated with integrating Medicare and Medicaid for dual eligible individuals, In Press 2023**	No change	No statistically significant change in hospital admissions in the integration vs. comparison cohorts.
**Keohane et al., Aligning Medicaid and Medicare Advantage Managed Care Plans for Dual-Eligible Beneficiaries, 2021**	Inconsistent/mixed	For 65+ duals, every 10%-point increase in aligned D-SNP participation, there was a -0.3 (-0.6, -0.0, $p<0.05$) change in hospital admissions. For <65 duals, 10% increase in aligned D-SNP was associated with a 0.2 (0.1, 0.4, $p<0.01$) increase in hospital admissions per month per 100 beneficiaries.
**Feng et al., Comparing Outcomes for Dual Eligible Beneficiaries in Integrated Care: Final Report, 2021**	Increased	Beneficiaries in FIDE-SNPs were more likely to be hospitalized than beneficiaries in non-integrated MA plans in 2015 (OR = 1.241; $p < 0.001$).

© 2024 Roberts et al. *JAMA Health Forum.*

**Kim et al, Comparing Care for Dual-Eligibles Across Coverage Models: Empirical Evidence from Oregon, 2019**	No change	Odds of having any hospitalization in a quarter in the aligned group was not statistically different (2.77% lower) than that for the FFS/FFS unaligned group from 2011 to 2014. Number of hospitalizations was statistically significantly lower in 2013 as compared to 2011 for the aligned plan versus FFS/FFS non-aligned plan by 2.43%, but there was no significant change in the other years in the model.
**Anderson et al, Effects of Integrating Care for Medicare-Medicaid Dually Eligible Seniors in Minnesota, 2020**	Decreased	Estimate of MSHO effect of any hospitalization was significantly lower in MSHO vs. MSC+ with coefficient of -0.104 (-0.111, -0.096, P<0.001), representing a 10.4 percentage point lower probability of hospital admission. Results hold after applying Oster's method to generate bias-adjusted estimates of the effects of MHSO.

© 2024 Roberts et al. *JAMA Health Forum.*

Emergency Department Visits

Study Author, Study Title, and Year	Findings regarding ED visits	Free text notes (succinct summary of findings)
**Chapin et al, Program of All-inclusive Care for the Elderly (PACE) Medicaid Cost-Benefit Study, 2013**	No change	No significant differences in PACE relative to HCBS/FE or NF (0.68 vs. 0.74 vs. 0.66 ER visits, respectively, per person/year).
**Feng et al., Comparing Outcomes for Dual Eligible Beneficiaries in Integrated Care: Final Report, 2021**	Decreased	Compared to beneficiaries in non-integrated MA plans, those in PACE were less likely to visit the ED in 2015 (OR = 0.523; $p < 0.001$).
**Griffin et al., MyCare Ohio: Third Evaluation Report, October 2023**	Increased	Over approximately 6.5 years, the monthly probability of any ED visit increased by 1.12 percentage points in the treatment vs. comparison groups.
**Holladay et al., Illinois Medicare-Medicaid Alignment Initiative: Third Evaluation Report, November 2022**	Increased	Over approximately 5 years, the monthly probability of any ED visit increased by 0.15 percentage points in the treatment vs. comparison groups.
**Howard et al., South Carolina Healthy Connections Prime Third Evaluation Report, December 2023**	No change	Over approximately 6 years, the monthly probability of an ED visit did not change significantly in the treatment vs. comparison groups.
**Gattine et al., Financial Alignment Initiative Massachusetts One Care: Preliminary Fifth Evaluation Report**	No change	Over approximately 6 years, demonstration was not associated with a statistically significant change in the monthly probability of any emergency department (ED) visits.
**Holladay et al., Financial Alignment Initiative Michigan MI Health Link Second Evaluation Report, March 2022**	No change	Over approximately 4 years, the monthly probability of an ED visit did not change significantly in the treatment vs. comparison groups.
**Gattine et al. Rhode Island Integrated Care Initiative: Third Evaluation Report, December 2023**	No Change	Over 4.5 years, the monthly probability of an ED visit did not change significantly in the treatment vs. comparison groups.
**Griffin et al., Dual Eligible Integrated Care Demonstration: Preliminary Third Evaluation Report, December 2023**	Increased	Over approximately 6 years, the monthly probability of an ED visit increased by 0.29 percentage points in the treatment vs. comparison groups, representing a 5.6% increase in the probability of any ED

© 2024 Roberts et al. *JAMA Health Forum.*

		visit in the demonstration group compared to the monthly average predicted probability for the comparison group.
**Caswell et al., Do Integrated Care Models for Dual Medicare-Medicaid Enrollees Work? Evidence from Massachusetts' One Care Financial Alignment Demonstration (In press, 2023)**	Decreased	The monthly probability of emergency department use is lower by 0.46 percentage points for the treatment group—i.e., just below the age cutoff. In relative terms, this equates to a 7.9 percent decrease using a base monthly utilization rate of 5.8 percent. However, this discontinuity is imprecisely estimated. Results for emergency department days per month were similar in sign and magnitude but were marginally statistically insignificant (p= 0.121).
**Chen et al, Early evidence from South Carolina's Medicare-Medicaid dual-eligible financial alignment initiative: an observational study to understand who enrolled, and whether the program improved health, 2018**	No change	No differential change in avoidable ED visits after 5 quarters between program participants and eligible non-participants.
**Graham et al., Beneficiaries Respond To California's Program to Integrate Medicare, Medicaid, And Long-Term Services, 2018**	No change	CMC vs. control: 0.58 vs. 0.84 ED visits per person in last 6mo from survey date (not significant).
**Roberts et al., Changes in care associated with integrating Medicare and Medicaid for dual eligible individuals, In Press 2023**	No change	No statistically significant change in emergency department visits in the integration vs. comparison cohorts.
**Keohane et al., Aligning Medicaid and Medicare Advantage Managed Care Plans for Dual-Eligible Beneficiaries, 2021**	No change	For 65+ duals, for every 10%-point increase in aligned-DSNP participation in county, there was a -0.4 (-1.2, 0.3) change in ED visits per month per 100 beneficiaries. For <65 duals, 10%-point increase in aligned D-SNP was associated with a change of -0.2 (-0.6, 0.3) in ED visits per month per 100 beneficiaries.
**Feng et al., Comparing Outcomes for Dual Eligible Beneficiaries in Integrated Care: Final Report, 2021**	Increased	Beneficiaries in FIDE-SNPs were more likely to visit the ED at least once in 2015 compared to beneficiaries in non-integrated MA plans (OR = 1.141; p < 0.001).

© 2024 Roberts et al. *JAMA Health Forum.*

**Kim et al, Comparing Care for Dual-Eligibles Across Coverage Models: Empirical Evidence from Oregon, 2019**	Decreased	Odds of having any ED Visit in a quarter declined by 16.71% in the aligned group compared to the FFS/FFS unaligned group from 2011 to 2014. Significant decreases were present for 2012 and 2013 compared to 2011 as well (3.85% and 7.35% decrease, respectively). The number of ED visits declined by 10.2% in the aligned group compared to the unaligned group from 2011 to 2014, but such decreases were not significant in 2012 and 2013 as compared to 2011.
**Anderson et al, Effects of Integrating Care for Medicare-Medicaid Dually Eligible Seniors in Minnesota, 2020**	No change	MSHO estimate effect: -0.008 (-0.016, 0.000), p=0.064. Not statistically significant.

© 2024 Roberts et al. *JAMA Health Forum.*

Outpatient Visits (Excluding ED)

Study Author, Study Title, and Year	Findings regarding outpatient visits/preventive services	Free text notes (succinct summary of findings)
**Griffin et al., MyCare Ohio: Third Evaluation Report, October 2023**	No change	Over approximately 6.5 years, the monthly number of physician E&M visits did not change significantly between the treatment vs. comparison groups.
**Holladay et al., Illinois Medicare-Medicaid Alignment Initiative: Third Evaluation Report, November 2022**	Increased	Over approximately 5 years, the monthly number of physician E&M visits increased by 148.93 visits per 1000 beneficiaries.
**Howard et al., South Carolina Healthy Connections Prime Third Evaluation Report, December 2023**	No change	Over approximately 6 years, the number of physician E&M visits did not change significantly between the treatment vs. comparison groups.
**Gattine et al., Financial Alignment Initiative Massachusetts One Care: Preliminary Fifth Evaluation Report**	Increased	Over approximately 6 years, demonstration was associated with an increase of 87.2 monthly number of physician evaluation and management visits per 1,000 beneficiaries in the treatment vs. comparison groups.
**Holladay et al., Financial Alignment Initiative Michigan MI Health Link Second Evaluation Report, March 2022**	Increased	Over approximately 4 years, the count of physician E&M visits increased by 0.08 per beneficiary per month in the treatment vs. comparison groups.
**Gattine et al. Rhode Island Integrated Care Initiative: Third Evaluation Report, December 2023**	Increased	Over 4.5 years, the monthly number of physician E&M visits increased to a greater extent (by 29.96 monthly visits per 1,000 beneficiaries) in the treatment vs. comparison groups.
**Griffin et al., Texas Dual Eligible Integrated Care Demonstration: Preliminary Third Evaluation Report, December 2023**	No change	Over approximately 6 years, the change in the monthly number of physician E&M visits did not differ significantly in the treatment vs. comparison groups.
**Caswell et al., Do Integrated Care Models for Dual Medicare-Medicaid Enrollees Work? Evidence from**	No change	No statistically significant discontinuity in ambulatory care use (any or days per person-month) across the age eligibility threshold for OneCare.

**Massachusetts' One Care Financial Alignment Demonstration (In press, 2023)**		
**Graham et al., Beneficiaries Respond To California's Program to Integrate Medicare, Medicaid, And Long-Term Services, 2018**	No change	CMC vs. control: 3.3 vs. 3.2 PCP visits per person last 6mo from survey date (not significant). Also not significant: same day appointments, visit for specialists; getting referrals/approvals for specialists; and easy to get appointments with specialists.
**Meyers et al., Medicare and Medicaid Dual-Eligible Special Needs Plan Enrollment and Beneficiary-Reported Experiences with Care, 2023**	Not examined	Note: Study asked respondents about their experiences "getting appointments and care quickly," which is summarized under the patient-reported experience domain.
**Roberts et al., Changes in care associated with integrating Medicare and Medicaid for dual eligible individuals, In Press 2023**	No change	No statistically significant change in outpatient physician visits in the integration vs. comparison cohorts.
**Kim et al, Comparing Care for Dual-Eligibles Across Coverage Models: Empirical Evidence from Oregon, 2019**	Increased	Odds of having a primary care visit in a quarter increased by 20.25% in the aligned group compared to the FFS/FFS unaligned group from 2011 to 2014. Such increases were not significant in 2012 and 2013 as compared to 2011. Number of primary care visits in a quarter also increased by 15.28% in the aligned group compared to the unaligned group from 2011 to 2014 (and 6.99% in the aligned group compared to FFS/FFS group from 2011 to 2013). The increase in PCP visits was not significant in 2012 compared to 2011.
**Anderson et al, Effects of Integrating Care for Medicare-Medicaid Dually Eligible Seniors in Minnesota, 2020**	Increased	Estimate of MSHO effect of any PCP visit was significantly higher in MSHO vs. MSC+ with coefficient of 0.141 (0.134, 0.149, P<0.001), implying that the likelihood of having a PCP visit was 14.1 percentage points higher in MSHO vs. MSC+. Results hold after applying Oster's method to generate bias-adjusted estimates of the effects of MHSO.

© 2024 Roberts et al. *JAMA Health Forum.*

Post-Acute Skilled Nursing Facility Stay

**Study Author, Study Title, and Year**	**Findings regarding post-acute SNF stays**	**Free text notes (succinct summary of findings)**
**Griffin et al., MyCare Ohio: Third Evaluation Report, October 2023**	Decreased	Over approximately 6.5 years, the monthly probability of a SNF admission decreased by 0.28 percentage points in the treatment vs. comparison groups.
**Holladay et al., Illinois Medicare-Medicaid Alignment Initiative: Third Evaluation Report, November 2022**	Increased	Over approximately 5 years, the monthly probability of a SNF admission increased by 0.22 percentage points in the treatment vs. comparison groups.
**Howard et al., South Carolina Healthy Connections Prime Third Evaluation Report, December 2023**	Decreased	Over approximately 6 years, the monthly probability of a SNF admission decreased by 0.25 percentage points in the treatment vs. comparison groups.
**Gattine et al., Financial Alignment Initiative Massachusetts One Care: Preliminary Fifth Evaluation Report**	Increased	Over approximately 6 years, demonstration was associated with a 0.04 percentage point increase in the probability of any skilled nursing facility (SNF) admission in the treatment vs. comparison groups.
**Holladay et al., Financial Alignment Initiative Michigan MI Health Link Second Evaluation Report, March 2022**	No change	Over approximately 4 years, the probability of SNF admission did not change significantly between the treatment vs. comparison groups.
**Gattine et al. Rhode Island Integrated Care Initiative: Combined FThird Evaluation Report, December 2023**	No change	Over approximately 4.5 years, the monthly probability of SNF admissions did not change significantly in the treatment vs. comparison groups.
**Griffin et al., Texas Dual Eligible Integrated Care Demonstration: Preliminary Third Evaluation Report, December 2023**	Decreased	Over approximately 6 years, the monthly probability of SNF use decreased by 0.17 percentage points in the treatment vs. comparison groups, corresponding to a relative decrease of 11.4%.
**Caswell et al., Do Integrated Care Models for Dual Medicare-Medicaid Enrollees Work? Evidence from Massachusetts' One Care Financial**	No change	No statistically significant discontinuity in the probability of SNF admissions across the age eligibility threshold for OneCare.

© 2024 Roberts et al. *JAMA Health Forum.*

**Alignment Demonstration (In press, 2023)**		
**Chen et al, Early evidence from South Carolina's Medicare-Medicaid dual-eligible financial alignment initiative: an observational study to understand who enrolled, and whether the program improved health, 2018**	No change	No differential change in SNF visits after 5 quarters between program participants and eligible non-participants. However, event study graphs suggest there were differential pre-trends for this outcome.
**Roberts et al., Changes in care associated with integrating Medicare and Medicaid for dual eligible individuals, In Press 2023**	No change	No statistically significant change in SNF stays in the integration vs. comparison cohorts.
**Jen Associates, Massachusetts Senior Care Option 2005-2010 Impact on Enrollees: Nursing Home Entry Utilization August 14, 2013**	No change	Null effects.

© 2024 Roberts et al. *JAMA Health Forum.*

Care Coordination (e.g., follow-up care after hospital stays)

Study Author, Study Title, and Year	Findings regarding care coordination	Free text notes (succinct summary of findings)
**Griffin et al., MyCare Ohio: Third Evaluation Report, October 2023**	Increased	Over approximately 6.5 years, the probability of 30-day follow-up after a mental health hospital discharge increased by 5.95 percentage points in the treatment vs. comparison groups.
**Holladay et al., Illinois Medicare-Medicaid Alignment Initiative: Third Evaluation Report, November 2022**	No change	Over approximately 5 years, the probability of 30-day follow-up after a mental health hospital discharge did not change significantly between the treatment vs. comparison groups.
**Howard et al., South Carolina Healthy Connections Prime Third Evaluation Report, December 2023**	No change	Over approximately 6 years, the probability of a follow-up visit after a mental health hospital discharge did not change significantly in the treatment vs. comparison groups.
**Gattine et al., Financial Alignment Initiative Massachusetts One Care: Preliminary Fifth Evaluation Report**	No change	Over approximately 6 years, no significant change in the probability of 30-day follow-up after mental health discharge was observed in the treatment vs. comparison groups
**Holladay et al., Financial Alignment Initiative Michigan MI Health Link Second Evaluation Report, March 2022**	No change	Over approximately 4 years, the probability of a follow-up visit after a mental health hospital discharge did not change significantly in the treatment vs. comparison groups.
**Gattine et al. Rhode Island Integrated Care Initiative: Third Evaluation Report, December 2023**	No change	Over 4.5 years, the probability of a follow-up visit after a mental health hospital discharge did not change significantly in the treatment vs. comparison groups.
**Griffin et al., Texas Dual Eligible Integrated Care Demonstration: Preliminary Third Evaluation Report, December 2023**	No change	Over approximately 6 years, the probability of a follow-up visit after a mental health hospital discharge did not change significantly in the treatment vs. comparison groups.
**Graham et al., Beneficiaries Respond To California's Program to Integrate Medicare, Medicaid, And Long-Term Services, 2018**	No change	CMC vs. control: No statistically significant difference in seeing any care coordinator (25.3% vs. 24%, respectively), getting all the care coordination services you need (68.2% vs. 70.1%), or receiving "important information about your medical history or treatment" (41.8% vs. 41.9%).

© 2024 Roberts et al. *JAMA Health Forum.*

**Meyers et al., Medicare and Medicaid Dual-Eligible Special Needs Plan Enrollment and Beneficiary-Reported Experiences with Care, 2023**	Not examined	Note: Study asked respondents about their experiences with care coordination, which is summarized under the patient-reported experience domain.
**Roberts et al., Changes in care associated with integrating Medicare and Medicaid for dual eligible individuals, In Press 2023**	No change	No statistically significant change in the probability of a follow-up outpatient visit within 14 days of a hospital stay in the integration vs. comparison cohorts.

© 2024 Roberts et al. *JAMA Health Forum.*

Hospital Admissions for Potentially Preventable Conditions (e.g., admissions for ambulatory care-sensitive conditions)

Study Author, Study Title, and Year	Findings re: potentially preventable admissions	Free text notes (succinct summary of findings)
**Griffin et al., MyCare Ohio: Third Evaluation Report, October 2023**	No change	Over approximately 6.5 years, the monthly number of hospitalizations for ambulatory care-sensitive conditions did not change significantly between the treatment vs. comparison groups.
**Holladay et al., Illinois Medicare-Medicaid Alignment Initiative: Third Evaluation Report, November 2022**	No change	Over approximately 5 years, the monthly number of hospitalizations for ambulatory care-sensitive conditions did not change significantly between the treatment vs. comparison groups.
**Howard et al., South Carolina Healthy Connections Prime Third Evaluation Report, December 2023**	Decreased	Over approximately 6 years, the probability of hospitalization for ambulatory care-sensitive conditions decreased by 0.16 percentage points in the treatment vs. comparison groups.
**Gattine et al., Financial Alignment Initiative Massachusetts One Care: Preliminary Fifth Evaluation Report**	Increased	Over approximately 6 years, the probability of any ambulatory care sensitive condition (ACSC) admission increased by 0.3 percentage points in the treatment vs. comparison groups.
**Holladay et al., Financial Alignment Initiative Michigan MI Health Link Second Evaluation Report, March 2022**	No change	Over approximately 4 years, the probability of hospitalization for ambulatory care-sensitive conditions did not change significantly in the treatment vs. comparison groups.
**Gattine et al. Rhode Island Integrated Care Initiative: Third Evaluation Report, December 2023**	No change	Over 4.5 years, the probability of hospital admission for ambulatory care-sensitive conditions did not change significantly in the treatment vs. comparison groups.
**Griffin et al., Texas Dual Eligible Integrated Care Demonstration: Preliminary Third Evaluation Report, December 2023**	No change	Over approximately 6 years, the probability of hospital admission for ambulatory care-sensitive conditions did not change significantly in the treatment vs. comparison groups.
**Caswell et al., Do Integrated Care Models for Dual Medicare-Medicaid Enrollees Work? Evidence from Massachusetts' One Care Financial**	No change	No statistically significant discontinuity in admissions for ambulatory care-sensitive conditions across the age eligibility threshold for OneCare.

© 2024 Roberts et al. *JAMA Health Forum.*

**Alignment Demonstration (In press, 2023)**		
**Roberts et al., Changes in care associated with integrating Medicare and Medicaid for dual eligible individuals, In Press 2023**	No change	No statistically significant change in hospital admissions for ambulatory care-sensitive conditions in the integration vs. comparison cohorts.
**Kim et al, Comparing Care for Dual-Eligibles Across Coverage Models: Empirical Evidence from Oregon, 2019**	Decreased	Odds of having a potentially avoidable hospitalization in a quarter decreased by 11.57% in the aligned group compared to the FFS/FFS unaligned group from 2011 to 2014. Such decreases were not significant in 2012 and 2013 as compared to 2011.

© 2024 Roberts et al. *JAMA Health Forum.*

Readmissions

**Study Author, Study Title, and Year**	**Findings regarding hospital readmissions**	**Free text notes (succinct summary of findings)**
**Griffin et al., MyCare Ohio: Third Evaluation Report, October 2023**	Decreased	Over approximately 6.5 years, the number of all-cause 30-day readmissions per 1000 discharges decreased by 24.07 (readmissions per 1000 discharges) in the treatment vs. comparison groups.
**Holladay et al., Illinois Medicare-Medicaid Alignment Initiative: Third Evaluation Report, November 2022**	No change	Over approximately 5 years, the number of all-cause 30-day readmissions per 1000 discharges did not change significantly between the treatment vs. comparison groups.
**Howard et al., South Carolina Healthy Connections Prime Third Evaluation Report, December 2023**	Decreased	Over approximately 6 years, the number of all-cause 30-day readmission per 1000 discharges decreased by 25.09 (readmission per 1000 discharges) in the treatment vs. comparison groups.
**Gattine et al., Financial Alignment Initiative Massachusetts One Care: Preliminary Fifth Evaluation Report**	Increased	Over approximately 6 years, in the treatment vs. comparison groups, there was an increase of approximately 13.5 all-cause 30-day readmissions per 1,000 discharges.
**Holladay et al., Financial Alignment Initiative Michigan MI Health Link Second Evaluation Report, March 2022**	No change	Over approximately 4 years, the number of all-cause 30-day readmissions did not change significantly between the treatment vs. comparison groups.
**Gattine et al. Rhode Island Integrated Care Initiative: Third Evaluation Report, December 2023**	No change	Over 4.5 years, the number of 30-day readmission per 1,000 discharges did not change significantly in the treatment vs. comparison groups.
**Griffin et al., Texas Dual Eligible Integrated Care Demonstration: Preliminary Third Evaluation Report, December 2023**	Increase	Over approximately 6 years, the cumulative demonstration effect was an increase of 9.88 in the number of all-cause 30-day readmissions per 1,000 discharges relative to the comparison group. This corresponded to a 3.7% increase in the number of all-cause 30-day readmissions among the demonstration group relative to the comparison group. The average number of all-cause 30-day readmissions decreased in both groups over time, but the decrease was smaller for the comparison group.

© 2024 Roberts et al. *JAMA Health Forum.*

**Caswell et al., Do Integrated Care Models for Dual Medicare-Medicaid Enrollees Work? Evidence from Massachusetts' One Care Financial Alignment Demonstration (In press, 2023)**	No change	No statistically significant difference in the probability of 30-day readmissions across the age eligibility threshold for OneCare.
**Kim et al, Comparing Care for Dual-Eligibles Across Coverage Models: Empirical Evidence from Oregon, 2019**	No change	Odds of all-cause 30-day hospital readmission was not significantly different for aligned group compared to unaligned group in 2014 as compared to 2011 (7.02% lower in 2014, 6.29% higher in 2013, .28% higher in 2012, none were significant).
**Jung et al, Integrated Medicare and Medicaid Managed Care and Rehospitalization of Dual Eligibles, 2015**	No change	No significant differences in 30-day all-cause readmission (adjusted odds ratio [AOR] of rehospitalization for SCO enrollees as compared to FFS enrollees was 1.13; 95% CI, 0.98-1.32).

© 2024 Roberts et al. *JAMA Health Forum.*

Patient Satisfaction and Experience with Care

Study Author, Study Title, and Year	Findings regarding patient satisfaction/ experience	Free text notes (succinct summary of findings)
**Griffin et al., MyCare Ohio: Third Evaluation Report, October 2023**	Not examined	Note: the evaluation reported changes in the proportion of MyCare Ohio enrollees rating their plan highly (9 or 10 on a 10-point scale). The report found increases over time in the proportion of enrollees rating their plan highly. However, this was a descriptive analysis of trends and not an analysis using a difference-in-differences framework.
**Snow et al. Financial Alignment Initiative New York Fully Integrated Duals Advantage for Individuals with Intellectual and Developmental Disabilities: Preliminary Third Evaluation Report. 2023.**	Not examined	Only descriptive analyses of certain satisfaction measures (e.g., grievances filed) were reported, but this was not presented alongside statistics for the comparison group.
**Holladay et al., Illinois Medicare-Medicaid Alignment Initiative: Third Evaluation Report, November 2022**	Not examined	Note: the evaluation reported changes in the proportion of Illinois MMP enrollees rating their plan highly (9 or 10 on a 10-point scale). The report found increases over time in the proportion of enrollees rating their plan highly. However, this was a descriptive analysis of trends and not an analysis using a difference-in-differences framework.
**Chepaitis et al., Virginia Commonwealth Coordinated Care Evaluation Report, Spring 2021**	Not examined	Note: the evaluation reported changes in the proportion of Virginia CCC enrollees rating their plan highly (9 or 10 on a 10-point scale). The report found increases over time in the proportion of enrollees rating their plan highly. However, this was a descriptive analysis of trends and not an analysis using a difference-in-differences framework.
**Howard et al., South Carolina Healthy Connections Prime Third Evaluation Report, December 2023**	Not examined	Note: the evaluation reported changes in the proportion of South Carolina Health Connections Prime MMP enrollees rating their plan highly (9 or 10 on a 10-point scale). The report found

		increases over time in the proportion of enrollees rating their plan highly. However, this was a descriptive analysis of trends and not an analysis using a difference-in-differences framework.
**Gattine et al., Financial Alignment Initiative Massachusetts One Care: Preliminary Fifth Evaluation Report**	Not examined	Descriptive analysis only
**Holladay et al., Financial Alignment Initiative Michigan MI Health Link Second Evaluation Report, March 2022**	Not examined	Note: the evaluation reported changes in the proportion of Michigan HealthLink MMP demonstration enrollees rating their plan highly (9 or 10 on a 10-point scale). The report found increases over time in the proportion of enrollees rating their plan highly. However, this was a descriptive analysis of trends and not an analysis using a difference-in-differences framework.
**Gattine et al. Rhode Island Integrated Care Initiative: Third Evaluation Report, December 2023**	Not examined	Descriptive analysis only
**Griffin et al., Texas Dual Eligible Integrated Care Demonstration: Preliminary Third Evaluation Report, December 2023**	Not examined	Note: the evaluation reported changes in the proportion of demonstration enrollees in Texas enrollees rating their plan highly (9 or 10 on a 10-point scale). The report found increases over time in the proportion of enrollees rating their plan highly. However, this was a descriptive analysis of trends and not an analysis using a difference-in-differences framework.
**Khatutsky et al, California Cal MediConnect Preliminary Third Evaluation Report, April 2023**	Not examined	Descriptive analysis only
**Graham et al., Beneficiaries Respond To California's Program to Integrate Medicare, Medicaid, And Long-Term Services, 2018**	No Change	CMC vs. control: Not statistically different in % "very satisfied" (73.3% vs. 68.9%, respectively), % excellent rating of quality of care (49.0% vs. 51.4%); but slightly more likely to experience delays or problem getting care/services/supplies (20.3% vs. 18.6%, $p<0.05$).
**Meyers et al., Medicare and Medicaid Dual-Eligible Special Needs Plan Enrollment and Beneficiary-Reported Experiences with Care, 2023**	Improved	Duals in FIDE-SNPs report higher ratings of care experience in a preponderance of domains of patient-reported care experience. Specifically, duals in FIDEs vs. non-D-SNP MA reported higher ratings on 5 domains: 1) overall rating of their plan (2.5 points on

© 2024 Roberts et al. *JAMA Health Forum.*

		10-point scale, [95% CI, 1.9, 3.1]), 2) prescription drug coverage (1.3 points [95% CI, 0.79 to 1.8]), 3) getting appointments and care quickly (1.1 points [95% CI, 0.0 to 2.2]), 4) customer service (1.5 points [0.3 to 2.7]), and 5) care quality rating (0.8 points [95% CI, 0.2 to 1.4]). However, duals in FIDEs vs. non-D-SNP MA reported lower ratings on 2 domains: 1) care coordination (-1.8 points [95% CI, -2.3 to -1.4]) and 2) getting needed prescription drugs (-1.9 points [95% CI, -3.4 to -0.3]).<br><br>Duals in FIDEs vs. coordinating only D-SNPs reported higher plan rating (0.8 [0.2 to 1.5]) and health care quality rating (0.7 [0.0 to 1.4]) but no differences in other measures.

© 2024 Roberts et al. *JAMA Health Forum.*

Mortality

Study Author, Study Title, and Year	Findings regarding mortality	Free text notes (succinct summary of findings)
**Chapin et al, Program of All-inclusive Care for the Elderly (PACE) Medicaid Cost-Benefit Study, 2013**	Inconsistent/mixed	PACE mortality was 52%, which was lower than those in NF (63%) but similar to those in HCBS/FE (50%).<br><br>Unmeasured differences between the NH group and the PACE group could introduce confounding results.
**Ghosh et al., Effects of PACE on Costs, Nursing Home Admissions, and Mortality 2006 - 2011, 2014**	Improved	PACE enrollees had consistently lower mortality than the matched comparison group that included HCBS waiver enrollees and NH entrants, with the difference being large and significant in each six-month period, ranging from 8-17 percentage points [p.p.] during each period after the first year through month 60. Relative to the comparison group that only included HCBS waiver enrollees, PACE enrollees also had lower mortality, but with smaller differences, ranging from 5-6 p.p. during each period after the first year through month 48. However, these findings could have been affected by unobserved confounding via unmeasured health and functional status.
**Feng et al., Comparing Outcomes for Dual Eligible Beneficiaries in Integrated Care: Final Report, 2021**	No Change	There was not a statistically significant difference in mortality between PACE enrollees and beneficiaries in non-integrated MA plans in 2015 (OR = 0.958; p = 0.062).
**Griffin et al., MyCare Ohio: Third Evaluation Report, October 2023**	Not examined	Mortality rates were not compared between treatment and comparison groups over the study period. (The only comparison was among MyCare Ohio-eligible individuals, between enrollees and non-enrollees, which primarily serves to test for selection into the program among those eligible, rather than an evaluation of a program effect.)
**Snow et al. Financial Alignment Initiative New York Fully Integrated Duals Advantage for Individuals with Intellectual and Developmental**	Not examined	Mortality rates were not compared between treatment and comparison groups over the study period. (The only comparison was among FIDA-IDD-eligible individuals, between enrollees and non-enrollees, which primarily serves to test for selection into the

© 2024 Roberts et al. *JAMA Health Forum.*

**Disabilities: Preliminary Third Evaluation Report. 2023.**		program among those eligible, rather than an evaluation of a program effect.)
**Holladay et al., Illinois Medicare-Medicaid Alignment Initiative: Third Evaluation Report, November 2022**	Not examined	Mortality rates were not compared between treatment and comparison groups over the study period. (The only comparison was among Illinois MMP-eligible individuals, between enrollees and non-enrollees, which primarily serves to test for selection into the program among those eligible, rather than an evaluation of a program effect.)
**Roberts et al., Changes in care associated with integrating Medicare and Medicaid for dual eligible individuals, In Press 2023**	Not examined	Differences in mortality were examined to test for potential unmeasured differences in risk factors preceding integration (i.e., selection bias at baseline). However, the study did not consider mortality to be an outcome of integration.
**Feng et al., Comparing Outcomes for Dual Eligible Beneficiaries in Integrated Care: Final Report, 2021**	Improved	Beneficiaries in FIDE-SNPs were significantly less likely to die in 2015 than beneficiaries in non-integrated MA plans (OR = 0.694; p < 0.001).<br><br>However, the study notes in reference to this finding that "low mortality risk might be attributable in part to unmeasured health characteristics of this population that were related to their relatively younger age but were not captured in the HCCs."
**Jen Associates, Massachusetts Senior Care Option 2005-2010 Impact on Enrollees: Nursing Home Entry Utilization August 14, 2013**	Improved	In the proportional hazards model, SCO enrollment was independently associated with a 17% reduction in the risk of death (Hazard ratio 0.83 for SCO enrollee, confidence limits 0.78 to 0.88, p value <.001).

© 2024 Roberts et al. *JAMA Health Forum.*

Self-Reported Health or Function

Study Author, Study Title, and Year	Findings regarding self-reported health or function	Free text notes (succinct summary of findings)
**Segelman et al., Transitioning from Community-Based to Institutional Long-term Care: Comparing 1915(c) Waiver and PACE Enrollee 2017**	Not examined	Technically, they examined cognitive impairment and overall function (ADLs) at time of NH admission, which was worse at time of NH admission for PACE enrollees than the HCBS waiver cohort; however, the fact that overall impairment of PACE enrollees is higher, it may be a positive for the PACE program since it could mean they kept people with more impairment out in community longer.

© 2024 Roberts et al. *JAMA Health Forum.*

Heterogeneity in Special Populations

**Study Author, Study Title, and Year**	Special populations examined (list here)	Free text notes (succinct summary of findings)
**Chapin et al, Program of All-inclusive Care for the Elderly (PACE) Medicaid Cost-Benefit Study, 2013**	(1) Needing nursing home level care.<br>(2) People with "greater cognitive needs." (LOC cognition score of 6 or 8).	(1) Overall findings apply to this subgroup.<br>(2) Further subgroup analysis is conducted for people with greater cognitive needs in PACE (4-year average Medicaid costs of $1786 per participant per month) also incurred lower Medicaid costs relative to those in NF ($3,044 per participant per month) though similar to HCBS/FE ($1783 per participant per month).
**Segelman et al., Transitioning from Community-Based to Institutional Long-term Care: Comparing 1915(c) Waiver and PACE Enrollee 2017**	Duals with LTSS use	Overall findings apply to subgroup.
**Ghosh et al., Effects of PACE on Costs, Nursing Home Admissions, and Mortality 2006 - 2011, 2014**	Duals with LTSS use	Overall findings apply to subgroup.
**Wieland et al, Does Medicaid Pay More to a Program of All-Inclusive Care for the Elderly (PACE) Than for Fee-for-Service Long-term Care?, 2013**	Duals with LTSS use	Overall findings apply to subgroup.
**Feng et al., Comparing Outcomes for Dual Eligible Beneficiaries in Integrated Care: Final Report, 2021**	Duals with LTSS use	Overall findings apply to subgroup.
**Griffin et al., MyCare Ohio: Third Evaluation Report, October 2023**	(1) Populations receiving LTSS;<br>(2) populations with serious	(1) Over approximately 6.5 years, the effect of the demonstration on LTSS users was an increase in the probability of SNF admissions and ED visits, a decrease in 30-day hospital re-admissions, and no significant change in hospital admissions, outpatient visits, or 30-day follow-up mental healthcare after a

© 2024 Roberts et al. *JAMA Health Forum.*

	persistent mental illness (SPMI)	mental health discharge (i.e., care coordination).<br><br>(2) Over approximately 6.5 years, the effect of the demonstration of populations with SPMI was a decrease in hospital admissions, SNF use, and 30-day hospital readmissions, and an increase in ED visits. Changes were not significant for outpatient visits and hospital admissions for ambulatory care sensitive conditions.
**Snow et al. Financial Alignment Initiative New York Fully Integrated Duals Advantage for Individuals with Intellectual and Developmental Disabilities: Preliminary Third Evaluation Report. 2023.**	The entire evaluation focused on individuals with intellectual and developmental disabilities -- the target population for New York's FIDA-IDD demonstration. No further analyses of subpopulations were conducted.	The entire evaluation focused on individuals with intellectual and developmental disabilities -- the target population for New York's FIDA-IDD demonstration. No further analyses of subpopulations were conducted.
**Holladay et al., Illinois Medicare-Medicaid Alignment Initiative: Third Evaluation Report, November 2022**	(1) Populations receiving LTSS; (2) populations with serious persistent mental illness (SPMI)	(1) Over approximately 5 years, the effect of the demonstration on LTSS users was an increase in the probability of SNF use, probability of inpatient admission, probability of an ED visit, number of outpatient visits, and probability of hospital admission for ambulatory care sensitive conditions. Changes were not significant for 30-day follow-up mental healthcare after a mental health discharge (i.e., care coordination) or 30-day hospital readmissions.<br><br>(2) Over approximately 5 years, the effect of the demonstration of populations with SPMI was an increase in SNF use and outpatient

© 2024 Roberts et al. *JAMA Health Forum.*

		visits. Changes were not significant for hospital admissions, ED visits, 30-day hospital readmission, and hospital admissions for ambulatory care sensitive conditions.
**Howard et al., South Carolina Healthy Connections Prime Third Evaluation Report, December 2023**	(1) Populations receiving LTSS; (2) populations with serious persistent mental illness (SPMI)	(1) Over approximately 6 years, the effect of the demonstration on LTSS users was a decrease in the monthly probability of any inpatient admission. Changes were not significant for outpatient visits, SNF use, ED visit use, 30-day follow up after a mental health hospitalization (i.e., care coordination), 30-day hospital readmissions, and hospital admissions for ambulatory care sensitive conditions.<br><br>(2) Over approximately 6 years, the effect of the demonstration of populations with SPMI was a decrease in the probability of hospital admissions, SNF use, ambulatory-care sensitive condition hospitalizations (overall and chronic); and an increase in ED use and number of preventable ED visits. Changes were not significant for outpatient visits and 30-day hospital readmission.
**Gattine et al., Financial Alignment Initiative Massachusetts One Care: Preliminary Fifth Evaluation Report**	(1) Populations receiving LTSS; (2) populations with serious persistent mental illness (SPMI)	(1) For beneficiaries with LTSS use (representing <1 percent of sample), effects of the demonstration included an increase in hospital admissions, SNF use, and 30-day hospital re-admissions. Changes were not significant for ED visits, outpatient visits, 30-day follow up after mental health hospitalization (i.e., care coordination), and hospital admissions for ambulatory care sensitive conditions.<br><br>(2) For those with SPMI (representing about 65 percent of the sample), effects included an increase in SNF use, outpatient visits, and 30-day hospital readmissions. Changes were not significant for hospital admissions, ED visits, and hospital admissions for ambulatory care sensitive conditions.
**Holladay et al., Financial Alignment Initiative Michigan MI Health Link**	(1) Populations receiving LTSS;	(1) Over approximately 4 years, the effect of the demonstration in populations with SPMI was a decrease in hospital admissions and

© 2024 Roberts et al. *JAMA Health Forum.*

**Second Evaluation Report, March 2022**	(2) populations with serious persistent mental illness (SPMI)	an increase in outpatient visits. Changes were not significant for SNF use, ED visits, hospital readmission, and hospital admission for ambulatory care sensitive conditions.<br><br>(2) Over approximately 4 years, the effect of the demonstration among LTSS users was a decrease in hospital admissions, ED visits, and hospital admissions for ambulatory care sensitive conditions. Changes were not significant for SNF use, outpatient visits, 30-day follow up after mental health hospitalization (i.e., care coordination), and 30-day hospital readmission.
**Gattine et al. Rhode Island Integrated Care Initiative: Third Evaluation Report, December 2023**	(1) Populations receiving LTSS; (2) populations with serious persistent mental illness (SPMI)	(1) Over 4.5 years, the demonstration was associated with an increase in overall ambulatory care sensitive conditions hospitalizations, and a decrease in ED visits and preventable ED visits. Changes were not significant for hospital admissions, outpatient visits, SNF use, 30-day follow up after mental health hospitalization, 30-day hospital readmission, and hospital admissions for chronic ambulatory care sensitive conditions.<br><br>(2) The demonstration in populations with SPMI was associated with insignificant changes in hospital admission, ED visits, SNF use, outpatient visits, preventable ED visits, 30-day hospital readmission, and hospital admission for ambulatory care sensitive conditions.
**Griffin et al., Texas Dual Eligible Integrated Care Demonstration Preliminary Third Evaluation Report, December 2023**	(1) Populations receiving LTSS; (2) populations with serious persistent mental illness (SPMI)	(1) Over approximately 6 years, the demonstration in LTSS users was associated with insignificant changes in the probability of ED visits, hospital admissions, SNF use, outpatient visits, 30-day follow up after mental health hospitalization (i.e., care coordination), 30-day hospital readmission, and hospital admission for ambulatory care sensitive conditions (overall and chronic).<br><br>(2) Over approximately 6 years, the demonstration in populations with SPMI was associated with insignificant changes in SNF use, ED visits, hospital admissions, outpatient visits, 30-day hospital

© 2024 Roberts et al. *JAMA Health Forum.*

		readmission, and hospital admission for ambulatory care sensitive conditions.
**Roberts et al., Changes in care associated with integrating Medicare and Medicaid for dual eligible individuals, In Press 2023**	Separate analyses were conducted among dual eligibles who qualified for nursing home-level care at baseline (e.g., HCBS recipients or residents of nursing homes at baseline).	Among individuals who received HCBS (via an HCBS waiver program) at baseline, integration was associated with differential increases in HCBS use, but no consistent changes in other outcomes, in each post-integration time period.<br><br>Among individuals who resided in a nursing home at baseline, integration was associated with differential increases in home health care use, but not consistent changes in other outcomes, in each post-integration period.
**Keohane et al., Aligning Medicaid and Medicare Advantage Managed Care Plans for Dual-Eligible Beneficiaries, 2021**	Duals with LTSS use	No significant effect of exposure to changes in aligned plan penetration on utilization (probability of being an HCBS user, hospital admissions, or prescription drugs) among the duals with LTSS needs under 65 and 65+. NH users are not examined with LTSS breakdown, as all NH residents use LTSS. For ED visits, Keohane et al found that increased aligned plan penetration was associated with a marginally statistically significant reduction in ED visits (-0.8 visits per 100 beneficiaries per month for each 10-percentage point increase in aligned plan penetration, 95% CI: -1.7 to 0.0). However, this reduction was only observed among LTSS users ages 65 and older and not among LTSS users under age 65. Thus, we characterize the overall effect on ED visits in this subgroup as inconsistent.

© 2024 Roberts et al. *JAMA Health Forum.*
