# Supplementary material for: Quality, Spending, Utilization, and Outcomes Among Dual-Eligible Medicare-Medicaid Beneficiaries in Integrated Care Programs: A Systematic Review
Source: JAMA Health Forum. 2024 Jul 19;5(7):e242187. doi: 10.1001/jamahealthforum.2024.2187 (PMC11259897; doi:10.1001/jamahealthforum.2024.2187)
Supplement: Supplement 2. — Data Sharing Statement. [file jamahealthforum-e242187-s002.pdf]

Data Sharing Statement

Roberts. Quality, Spending, Utilization, and Outcomes Among Dual-Eligible Medicare-Medicaid Beneficiaries in Integrated Care Programs. *JAMA Health Forum*. Published July 19, 2024. doi:10.1001/jamahealthforum.2024.2187

Data
Data available: Yes
Data types: Other (please specify)
Additional Information: Data extractions from the systematic review are included in full in the online supplement
How to access data: Data extractions from the systematic review are included in full in the online supplement
When available: With publication

Supporting Documents
Document types: None

Additional Information
Who can access the data: Data extractions from the systematic review are included in full in the online supplement
Types of analyses: Systematic Review
Mechanisms of data availability: Online supplement
Any additional restrictions: NA
